# Aging diminishes the resistance of AO rats to EAE: putative role of enhanced generation of GM-CSF Expressing CD4+ T cells in aged rats

**DOI:** 10.1186/s12979-015-0044-x

**Published:** 2015-10-06

**Authors:** Zorica Stojić-Vukanić, Mirjana Nacka-Aleksić, Ivan Pilipović, Ivana Vujnović, Veljko Blagojević, Duško Kosec, Mirjana Dimitrijević, Gordana Leposavić

**Affiliations:** Department of Microbiology and Immunology, Faculty of Pharmacy, University of Belgrade, 450 Vojvode Stepe, 11221 Belgrade, Serbia; Department of Physiology, Faculty of Pharmacy, University of Belgrade, 450 Vojvode Stepe, 11221 Belgrade, Serbia; Immunology Research Centre “Branislav Janković”, Institute of Virology, Vaccines and Sera “Torlak”, 458 Vojvode Stepe, 11221 Belgrade, Serbia

**Keywords:** AO rats, Aging, EAE, GM-CSF

## Abstract

**Background:**

Aging influences immune response and susceptibility to EAE in a strain specific manner. The study was designed to examine influence of aging on EAE induction in Albino Oxford (AO) rats.

**Results:**

Differently from 3-month-old (young) rats, which were resistant to EAE induction, the majority of aged (24-26-month-old) rats developed mild chronic form of EAE. On 16^th^ day post-immunization, when in aged rats the neurological deficit reached plateau, more mononuclear cells, including CD4+ T lymphocytes was retrieved from spinal cord of aged than young rats. The frequencies of IL-17+ and GM-CSF+ cells within spinal cord infiltrating CD4+ lymphocytes were greater in aged rats. To their increased frequency contributed the expansion of GM-CSF + IL-17 + IFN-γ+ cells, which are highly pathogenic in mice. The expression of the cytokines (IL-1β and IL-23/p19) driving GM-CSF + IL-17 + IFN-γ + cell differentiation in mice was also augmented in aged rat spinal cord mononuclear cells. Additionally, in aged rat spinal cord the expansion of GM-CSF + IL-17-IFN-γ- CD4+ T lymphocytes was found. Consistently, the expression of mRNAs for IL-3, the cytokine exhibiting the same expression pattern as GM-CSF, and IL-7, the cytokine driving differentiation of GM-CSF + IL-17-IFN-γ- CD4 + lymphocytes in mice, was upregulated in aged rat spinal cord mononuclear cells, and the tissue, respectively. This was in accordance with the enhanced generation of the brain antigen-specific GM-CSF+ CD4+ lymphocytes in aged rat draining lymph nodes, as suggested by (i) the higher frequency of GM-CSF+ cells (reflecting the expansion of IL-17-IFN-γ- cells) within their CD4+ lymphocytes and (ii) the upregulated GM-CSF and IL-3 mRNA expression in fresh CD4+ lymphocytes and MBP-stimulated draining lymph node cells and IL-7 mRNA in lymph node tissue from aged rats. In agreement with the upregulated GM-CSF expression in aged rats, strikingly more CD11b + CD45^int^ (activated microglia) and CD45^hi^ (mainly proinflammatory dendritic cells and macrophages) cells was retrieved from aged than young rat spinal cord. Besides, expression of mRNA for SOCS1, a negative regulator of proinflammatory cytokine expression in innate immunity cells, was downregulated in aged rat spinal cord mononuclear cells.

**Conclusions:**

The study revealed that aging may overcome genetic resistance to EAE, and indicated the cellular and molecular mechanisms contributing to this phenomenon in AO rats.

**Electronic supplementary material:**

The online version of this article (doi:10.1186/s12979-015-0044-x) contains supplementary material, which is available to authorized users.

## Background

Multiple sclerosis (MS) is a complex inflammatory autoimmune disease of the central nervous system (CNS) with heterogeneous clinical, pathological and immunological phenotype that might better be described as a syndrome rather than a single disease entity. The etiology of MS is not well understood, but it is believed that myelin-specific CD4^+^ T cells play a central role in initiating and orchestrating CNS inflammation [1]. Their role has been studied extensively, principally by using experimental autoimmune encephalomyelitis (EAE), an animal model of MS. EAE can be actively induced in susceptible strains of mice and rats by immunization with either whole spinal cord homogenate or encephalitogenic proteins or peptides in adjuvants. Both Th1 and Th17 cells have been implicated in MS/EAE development [1, 2]. Thus, the role of CD4+ cells in MS/EAE pathogenesis has been called the Th1/Th17 paradigm [3]. However, there is a body of evidence indicating that Th1 and Th17 cytokines are dispensable for the development of EAE [4–7]. Additionally, granulocyte macrophage colony-stimulating factor (GM-CSF) is suggested to be the only known cytokine produced by T cells that is required for susceptibility to EAE [8]. Furthermore, the pathogenicity of autoreactive Th17 cells has been associated with their production of GM-CSF [8, 9]. Although Th1 cells are also shown to produce GM-CSF, they most likely do not represent an important source of GM-CSF in EAE [8]. Recently, IL-7-STAT5 signalling axis-induced CD4+ T cells are suggested to be the major source of GM-CSF in T cell-mediated neuroinflammation [10]. Besides, they are thought to represent a new Th subset with a distinct differentiation program and cytokine production profile [10]. This subset has been designated as Th-GM subset [10]. GM-CSF accelerates the release of bone marrow precursors that ultimately differentiate into the CNS-infiltrating dendritic cells and macrophages [11], and provides activation/expansion of cells belonging to the myeloid lineage, which in turn promote (i) Th-GM cell activation/differentiation and (ii) tissue destruction through release of various bioactive molecules [8, 9, 12, 13].

The MS typically begins between the ages of 20 and 40 years, whereas initial symptoms rarely occur before age of 10 years or after age of 60 years [14]. Data on the influence of aging on the incidence and clinical course of actively induced EAE are limited and inconsistent [15–21]. This inconsistency most likely could be ascribed to species and strain differences, differences in animal chronological (and possibly biological) age and immunization protocols. The discrepancy between data obtained in humans and in animal models, apart from differences in etiopathogenesis [22–24], could also reflect the fact that influence of aging on autoimmune neuroinflammation has been studied in inbred rodent strains, while, though there are human populations of relative genetic homogeneity (e.g. Amish), in most human populations there is significant genetic diversity [25]. Despite all the aforementioned, the investigations of influence of aging on EAE development in distinct rodent strains are important as they not only offer a window into the putative mechanisms underlying MS development, but also broaden our understanding of age-related immune changes.

Albino Oxford (AO) rats are relatively resistant to EAE induction [26]. More specifically, their resistance to EAE induction is not absolute, but quantitative, as minimal single cell infiltrate is regularly seen (independently on immunization protocol) in spinal cord of AO rats although they do not develop clinical signs of EAE [27]. Furthermore, resistance of AO rats to EAE is not due to their inability to recognize determinants of myelin basic protein (MBP) [26]. Their resistance is suggested to be related to the differences in immunoregulatory circuits affecting either antigen presenting cells or lymphocytes with regulatory properties [26]. Our recent studies showed that aging markedly affects phenotypic and functional characteristics of splenic myeloid dendritic cells from AO rats, and that this effect is strain-specific [28, 29]. In the rat, dendritic cell subset composition correlates with their susceptibility to autoimmune disease induction and Th polarization [30, 31]. The greater proportion of CD4-CD11b + OX62+ cells (producing large amount of proinflammatory cytokines) within dendritic cell population has been associated with a greater susceptibility to Th1/Th17-mediated diseases [30, 31]. In AO rats, age-related increase in the relative proportion of CD4-CD11b + OX62+ cells within splenic myeloid dendritic cells was found [28]. Consistently, *in vitro* LPS-activated splenic myeloid dendritic cells from aged AO rats expressed more TNF-α, IL-12, IL-6 and IL-23, and exhibited the enhanced Th1/Th17 driving capacity in co-cultures with allogeneic CD4+ lymphocytes, when compared with those cells from young strain-matched rats [28]. The previous findings seem to be particularly relevant in light of data indicating that brain tissue resident dendritic cells in steady state share a similar phenotype and genotype profiling with splenic dendritic cells, as both dendritic cell subsets have a common precursor as pre-dendritic cells or peripheral blood dendritic cells that are derived from the bone marrow [32, 33]. In agreement with the aforementioned data, our preliminary findings indicated that AO rat susceptibility to EAE increases with aging [28]. Consequently, we undertook a series of experiments in order to elucidate cellular and molecular mechanisms standing behind this phenomenon. For this purpose, phenotypic and functional characteristics of CD4+ T lymphocytes and antigen presenting cells from spinal cords and draining lymph nodes of young and aged AO rats were examined in inductive and effector phases of EAE.

## Results

### Aged AO rats immunized for EAE develop mild chronic disease

**Fig. 1:**
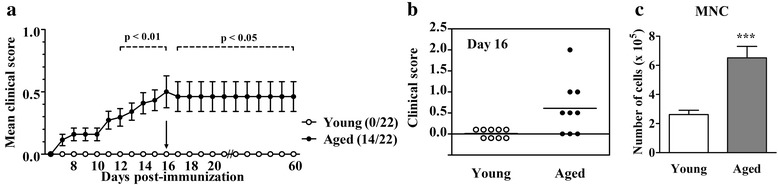
Aging diminishes resistance of AO rats to EAE development. (**a**) Aged and young AO rats were immunized with rat spinal cord homogenate in complete Freund’s adjuvant and co-injected with *Bordetella pertussis*. The clinical course of EAE was evaluated daily. Neurological signs of EAE were scored as indicated in the section Methods. Thirteen rats from each group were followed over a 60-day-long follow-up period in a preliminary experiment, whereas 9 rats were sacrificed on the 16^th^ d.p.i. (arrow in line graph) for analyses. Line graph indicates the daily neurological score of EAE in aged and young AO rats. The first numbers in the brackets indicate the number of rats with clinical EAE, whereas the second number (22) indicates the total number of immunized rats per group. (**b**) Scatter plot indicates the clinical score of EAE on the 16^th^ d.p.i. in rats used for analyses. Horizontal lines within the scatters represent the mean values. (**c**) Bar graph indicates the total number of mononuclear cells retrieved from spinal cords of aged and young rats on the 16^th^ d.p.i. Data represent mean values ± SEM (*n* = 9/group). Data shown are from one of two independent experiments with similar results. ***p < 0.001

Differently from young AO rats, which were resistant to the induction of clinical EAE, 14 animals out of 22 aged rats immunized for EAE (i.e. 6 rats from 9 rats sacrificed on the 16^th^ d.p.i. and 8 rats from 13 rats, which were followed until 60^th^ d.p.i.) exhibited mild signs of the disease (Fig. 1). In aged rats, which developed EAE, the clinical (neurological) score reached the plateau value between 15^th^ and the 16^th^ d.p.i. (Fig. 1). In agreement with the neurological findings, on the 16^th^ d.p.i. greater (p < 0.001) number of mononuclear cells was retrieved from aged compared with young rat spinal cord (Fig. 1).

### Aging increases the number of CD4+ TCRαβ + lymphocytes infiltrating the spinal cord of AO rats immunized for EAE

**Fig. 2:**
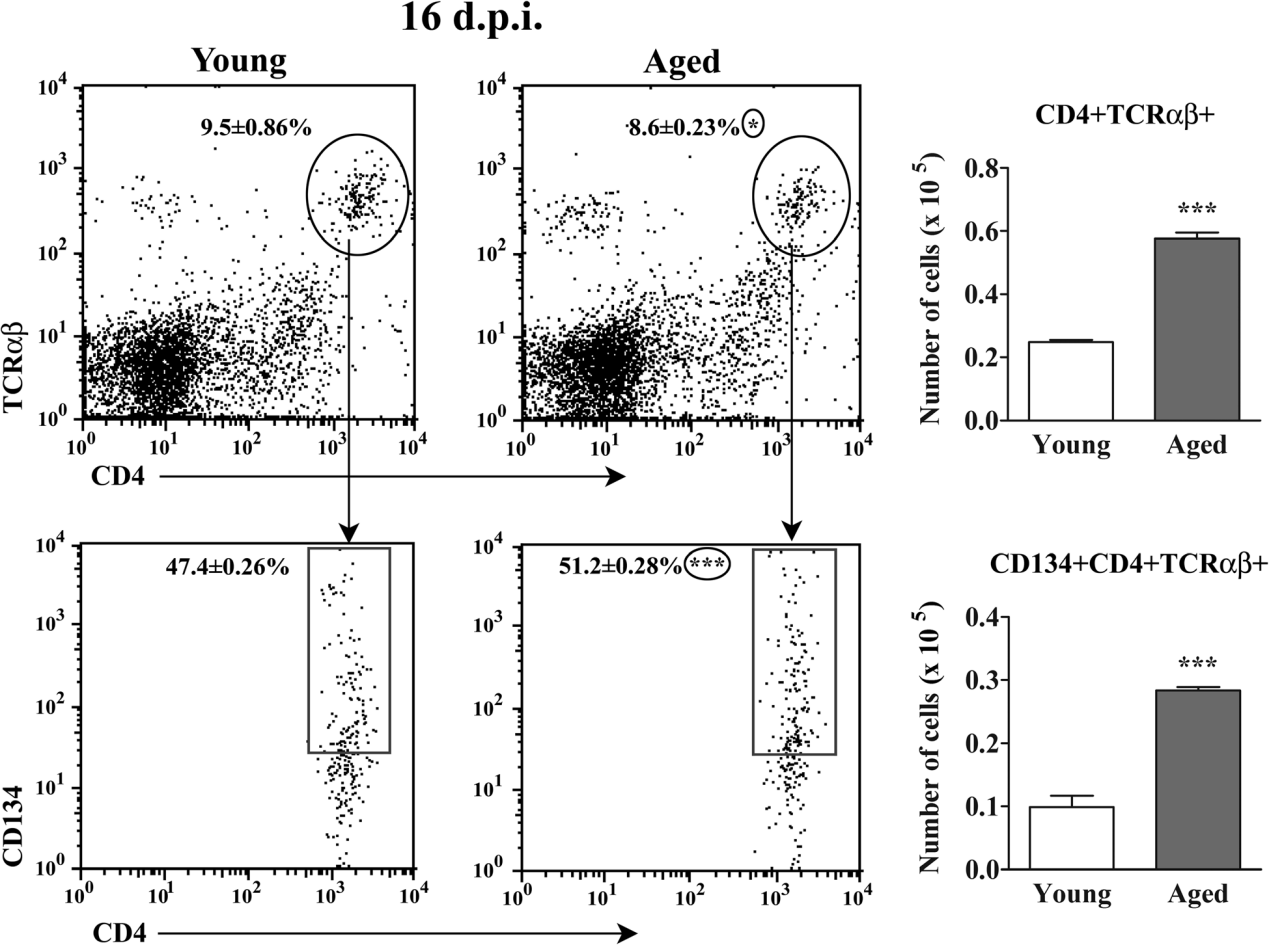
Aging increases the number of CD134 + CD4+ T cells in spinal cord of AO rats immunized for EAE. Lower flow cytometry dot plots show CD134 vs CD4 staining of lymphocytes retrieved from spinal cords of (left) young and (right) aged rats on the 16^th^ d.p.i. CD4+ TCRαβ + cells were gated as shown in the upper flow cytometry dot plots. Numbers in the flow cytometry dot plots represent the percentage of (upper dot plots) CD4+ TCRαβ + cells within spinal cord cells and (lower dot plots) CD134+ cells among CD4+ TCRαβ + lymphocytes. Bar graphs show the number of (upper) CD4+ TCRαβ + cells and (lower) CD134 + CD4+ TCRαβ + cells in young and aged rat spinal cords. All results are presented as means ± SEM (n = 9/group). Data are representative of one of two experiments with similar results. *p < 0.05; ***p < 0.001

The analysis of spinal cord mononuclear cells isolated on the 16^th^ d.p.i. revealed slightly lower (*p* < 0.05) frequency of CD4+ TCRαβ + lymphocytes (Fig. 2). However, despite the lower frequency of the spinal cord infiltrating CD4+ TCRαβ + lymphocytes, more (*p* < 0.001) the cells was retrived from aged than young rat spinal cord (Fig. 2). The greater number of CD4+ TCRαβ + lymphocytes in aged rat spinal cord did not reflect their better survival, as the frequency of apoptotic cells was comparable among CD4+ TCRαβ + cells from aged and young rats (Additional file 1: Figure S1).

Further analyses of the spinal cord infiltrating CD4+ TCRαβ + lymphocytes showed higher (*p* < 0.001) frequency and number of CD134+ cells, presumably reactivated cells [21, 34] in aged compared with young rats (Fig. 2).

#### Aging increases the frequency of IL-17+ cells among CD4+ T lymphocytes infiltrating the spinal cord of rats immunized for EAE

**Fig. 3:**
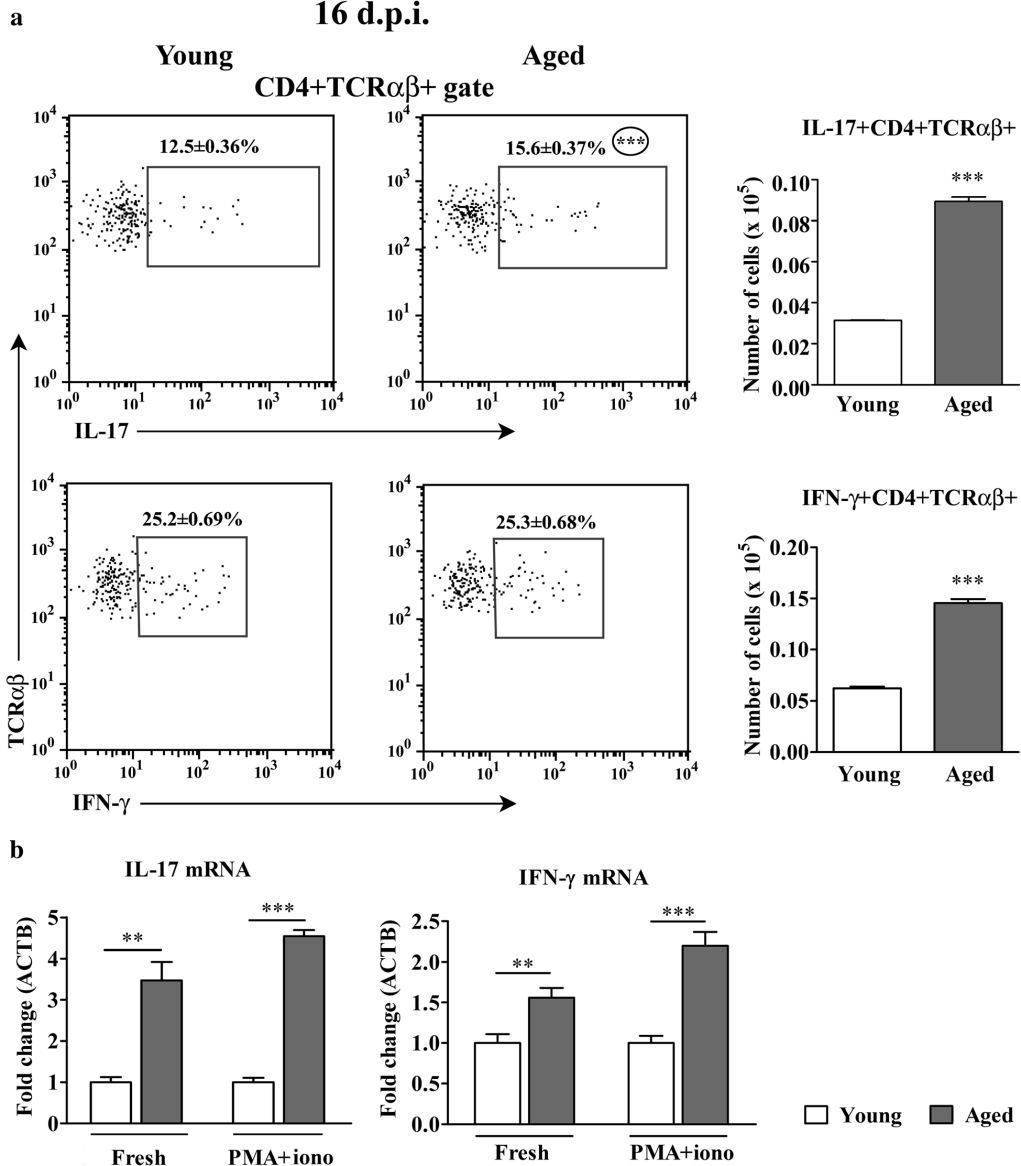
Aging increases the number of IL-17+ CD4+ T cells infiltrating the spinal cord of AO rats immunized for EAE. (Panel **a**) Flow cytometry dot plots indicate the expression of (upper) IL-17 and (lower) IFN-γ in CD4+ TCRαβ + lymphocytes from spinal cord of (left) young and (right) aged rats on the 16^th^ d.p.i. Numbers in the flow cytometry dot plots represent the percentage of cells in the indicated region. Bar graphs indicate the number of (upper) IL-17 + CD4+ TCRαβ + and (lower) IFN-γ + CD4+ TCRαβ + lymphocytes in spinal cords from young and aged rats. (Panel **b**) Bar graphs indicate the fold change in expression of mRNAs for IL-17 and IFN-γ in freshly isolated (Fresh) and PMA- and ionomycin-stimulated (PMA + iono) mononuclear spinal cord cells from aged relative to young rats on the 16^th^ d.p.i. as determined by RT-qPCR. Data are normalized to β-actin (ACTB). All results are presented as means ± SEM (*n* = 9/group). Data are representative of one of two experiments with similar results. **p < 0.01; ***p < 0.001

Considering the Th1/Th17 paradigm [3], the spinal cord infiltrating CD4+ TCRαβ + lymphocytes were also examined for the expression of IL-17 and IFN-γ. Flow cytometry analysis (FCA) revealed greater (*p* < 0.001) relative and absolute numbers of IL-17+ cells within the spinal cord infiltrating CD4+ TCRαβ + lymphocytes from aged compared with young rats (Fig. 3). In accordance with the previous findings, the expression of mRNA for IL-17, a Th17 signature cytokine, was greater in freshly isolated (p < 0.01) and PMA- and ionomycin-stimulated (*p* < 0.001) spinal cord mononuclear cells from aged rats compared with their younger counterparts (Fig. 3).

The frequency of IFN-γ + cells among CD4+ TCRαβ + lymphocytes infiltrating spinal cord was comparable between aged and young rats (Fig. 3). However, due to the greater cellularity of CD4+ TCRαβ + subset, the number of IFN-γ + cells was also greater (*p* < 0.001) in spinal cord from aged compared with young rats (Fig. 3). Although the frequency of IFN-γ + cells within CD4+ TCRαβ + lymphocytes was comparable between aged and young rats, the greater amount of mRNA for this cytokine was found in fresh (*p* < 0.01) and PMA- and ionomycin-stimulated (*p* < 0.001) spinal cord mononuclear cells from aged compared with young rats (Fig. 3).

#### Aging increases the frequency of GM-CSF+ cells among CD4+ T lymphocytes infiltrating the spinal cord of rats immunized for EAE

**Fig. 4:**
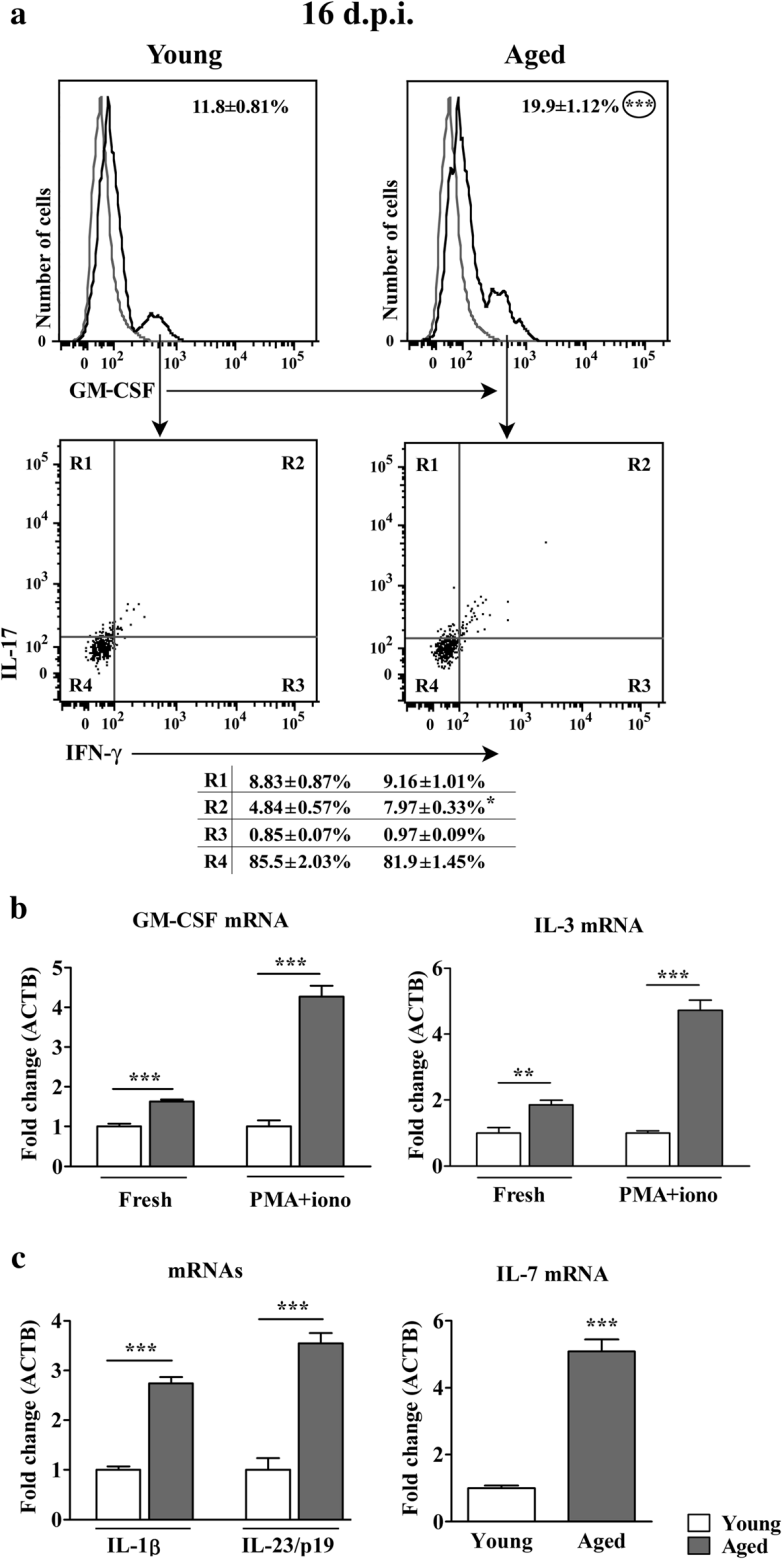
Aging increases the frequency of GM-CSF+ cells within CD4+ T lymphocytes infiltrating the spinal cord of AO rats immunized for EAE. (Panel **a**) Overlaid flow cytometry histograms show GM-CSF expression in CD4+ T cells isolated on the 16^th^ d.p.i. using MACS (described in detail in the section Methods) from spinal cords of (left) young and (right) aged rats. In the overlaid flow cytometry histograms left histograms (thin grey line) represent nonspecific binding of secondary antibody. Flow cytometry dot plots show IFN-γ vs IL-17 staining of GM-CSF+ CD4+ T cells retrieved from spinal cords of (left) young and (right) aged rats on the 16^th^ d.p.i. Numbers in the flow cytometry profiles represent the percentage of cells in the indicated region. (Panel **b**) Bar graphs indicate the fold change in expression of mRNAs for GM-CSF and IL-3 in freshly isolated (Fresh) and PMA- and ionomycin-stimulated (PMA + iono) mononuclear spinal cord cells from aged relative to young rats on the 16^th^ d.p.i. as determined by RT-qPCR. (Panel **c**) Bar graphs indicate the fold change in expression of mRNAs for IL-1β and IL-23/p19 in spinal cord mononuclear cells and IL-7 in spinal cord tissue from aged relative to young rats on the 16^th^ d.p.i. as determined by RT-qPCR. Data are normalized to β-actin (ACTB). All results are presented as means ± SEM (*n* = 9/group). The data, except for GM-CSF, are representative of one of two experiments with similar results. *p < 0.05; **p < 0.01; ***p < 0.001

Next, the frequency of cells producing GM-CSF, which is shown to play the central role in maintaining chronic neuroinflammation [8, 35, 36], among CD4+ T lymphocytes was examined. The frequency of GM-CSF+ cells among CD4+ T lymphocytes infiltrating spinal cord was increased (*p* < 0.001) in aged rats when compared with their younger counterparts (Fig. 4). Consistently, the expression of GM-CSF mRNA was upregulated (*p* < 0.001) in both fresh and PMA- and ionomycin-stimulated spinal cord mononuclear cells from aged rats compared with young ones (Fig. 4).

Next, considering recent findings suggesting that in neuroinflammation, apart from GM-CSF+ CD4+ T lymphocytes that do not express Th1, Th17 and Th2 signature cytokines, GM-CSF+ CD4+ T lymphocytes co-producing IL-17 in mice [10], and IFN-γ in humans [37] play significant pathogenic role, GM-CSF+ CD4+ T lymphocytes were examined for the production of IL-17 and IFN-γ using FCA. Irrespective of rat age, the majority of GM-CSF+ CD4+ spinal cord infiltrating T lymphocytes exhibited IL-17-IFN-γ- phenotype (Fig. 4). The frequency of these cells did not significantly differ within spinal cord infiltrating GM-CSF+ CD4+ T cells from aged and young rats (Fig. 4). Thus, since the frequency of GM-CSF+ cells was convincingly higher within CD4+ T lymphocytes from aged rat spinal cord, the frequency of GM-CSF + IL-17-IFN-γ- cells was also higher (16.30 ± 1.26 in aged vs 10.09 ± 0.91 in young rats; *p* < 0.01) among CD4+ T lymphocytes infiltrating the spinal cord of aged rats when compared with young ones. Next, the expression of mRNA for IL-4, Th2 signature cytokine, in spinal cord mononuclear cells was examined. Irrespective of rat age, the expression of IL-4 mRNA was below the limit of accurate quantification. Considering that in the mouse GM-CSF+ cells, which do not express Th1, Th17 and Th2 signature cytokines (termed Th-GM cells) express a similar pattern of GM-CSF and IL-3 production [10], IL-3 mRNA expression was examined in fresh and PMA- and ionomycin-stimulated spinal cord mononuclear cells. The expression of IL-3 mRNA was augmented in both fresh (*p* < 0.01) and PMA- and ionomycin-stimulated (*p* < 0.001) spinal cord mononuclear cells from aged rats compared with young ones (Fig. 4). This finding, in conjunction with the previous ones, could suggest the expansion of GM-CSF CD4+ T lymphocytes on the account of cells that resemble mouse Th-GM cells, in aged rats. Furthermore, within GM-CSF+ subset of CD4+ T lymphocytes, a significant proportion of cells produced either IL-17 only or both IL-17 and IFN-γ. A small subset of GM-CSF+ CD4+ T lymphocytes also produced IFN-γ (Fig. 4). The frequency of IL-17+ and IFN-γ + single positive cells within GM-CSF + CD4+ T lymphocytes infiltrating the spinal cord of aged and young rats was comparable, whereas that of IL-17 + IFN-γ + double positive cells was greater (*p* < 0.05) in aged rats (Fig. 4). Given that the frequency of all GM-CSF+ cells was greater among CD4+ T lymphocytes infiltrating the spinal cord of aged rats, the frequency of GM-CSF + IL-17 + IFN-γ + was also greater (*p* < 0.01) among these cells from aged (1.59 ± 0.08 %) compared with young rats (0.57 ± 0.01 %). In mice [38], differently from humans [37, 39], the triple cytokine-producing cells are derived from plastic Th17 cells, and their pathogenic propensity in EAE is related to GM-CSF production [38].

Considering that Th-GM cells in mice differentiate under the influence of IL-7-mediated STAT5 signaling [10], IL-7 mRNA expression was examined in spinal cord tissue. Indeed, the expression of mRNA for IL-7 was upregulated (p < 0.001) in aged compared with young animals (Fig. 4). This further corroborated the previous notion that GM-CSF + IL-17-IFN-γ- cells accumulating in aged rat spinal cord resemble mouse Th-GM cells.

Next, given that in the mouse the differentiation of IL-17 + IFN-γ + CD4+ T lymphocytes, whose pathogenicity is related to GM-CSF production, is driven by IL-1β and IL-23 [8, 9], the expression of mRNAs for these cytokines was measured in spinal cord mononuclear cells. The expression of mRNA for both the cytokines was also upregulated (*p* < 0.001) in aged compared with young animals (Fig. 4).

### Aging leads to the expansion of CD11b + CD45^hi^ cell population in spinal cord of rats immunized for EAE on the 16^th^ d.p.i.

**Fig. 5:**
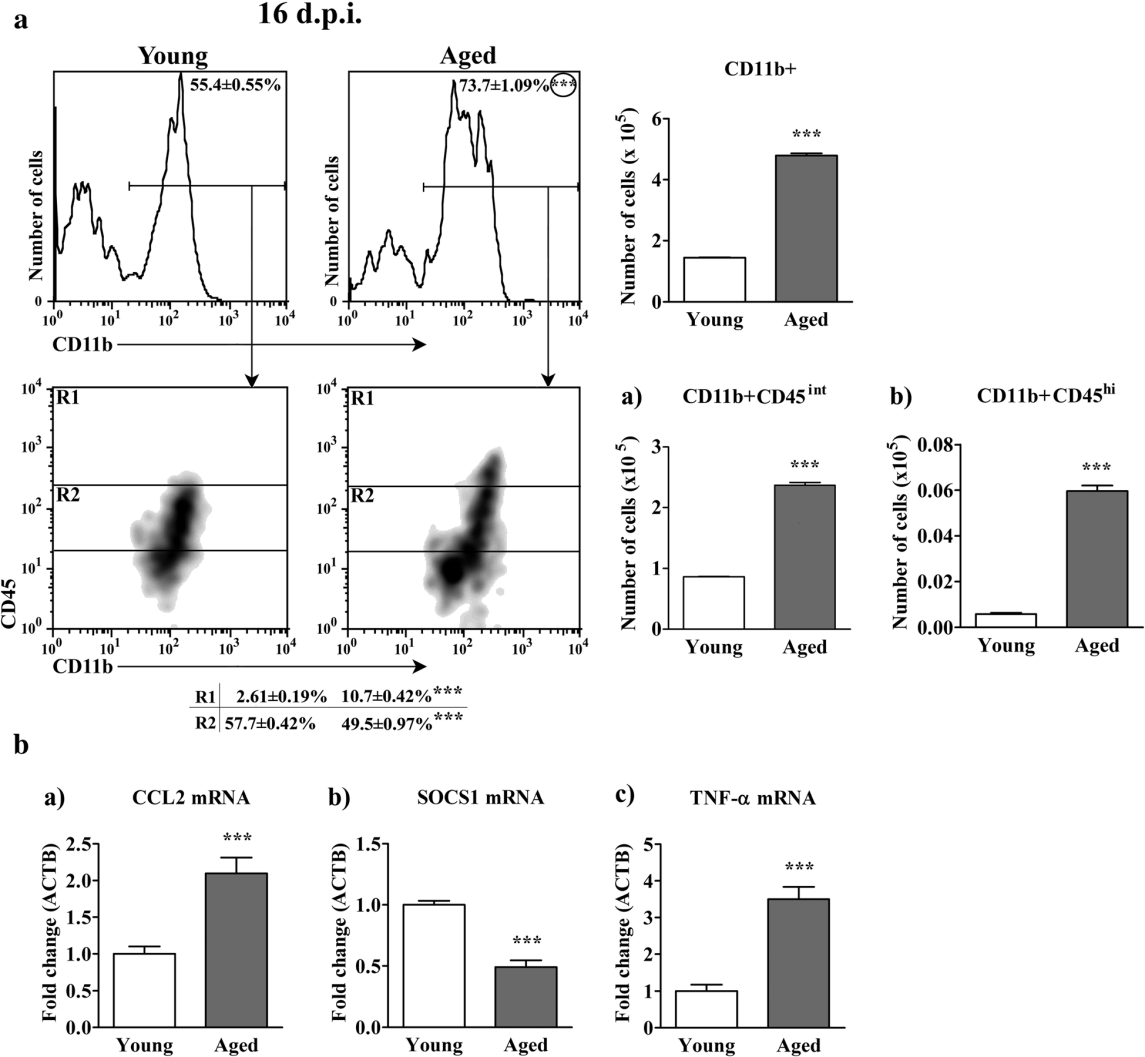
Aging increases the number of all CD11b + nonlymphoid cells, and CD45^hi^ CD11b + cells, recovered on the 16^th^ d.p.i. from AO rat spinal cord. (Panel **a**) Flow cytometry density plots indicate the expression of CD45 on CD11b + cells from spinal cord of (left) young and (right) aged rats on the 16^th^ d.p.i. CD11b + cells were gated as shown in the flow cytometry histograms. Numbers in the flow cytometry histograms represent the percentage of CD11b + cells. R1 = CD11b + CD45^hi^ cells; R2 = CD11b + CD45^int^ cells. Numbers in the table represent the percentage of cells in the indicated region (R). Bar graphs indicate the number of (upper) CD11b + and (lower left) CD11b + CD45^int^ and (lower right) CD11b + CD45^hi^ spinal cord mononuclear cells retrieved from young and aged rats. (Panel **b**) Bar graphs indicate the fold change in mRNA expression for (a) CCL2 in spinal cord tissue and (b) SOCS1 and (c) TNFα in spinal cord mononuclear cells from aged relative to young rats on the 16^th^ d.p.i. as determined by RT-qPCR. Data are normalized to β-actin (ACTB). All results are presented as means ± SEM (*n* = 9/group). Data are representative of one of two experiments with similar results. ***p < 0.001

Although CD4+ TCRαβ + lymphocytes were more numerous in spinal cord of aged rats, the expansion of CD11b + cell population mainly contributed to the greater number of mononuclear cells isolated from aged rats. Thus, the proportion of CD11b + cells within mononuclear cells isolated from aged rat spinal cord, and their number were greater (*p* < 0.001) when compared with young rats (Fig. 5). CD11b + spinal cord cell population encompasses resident microglial cells, monocyte-derived dendritic cells, also called inflammatory dendritic cells [12, 40], and blood-borne macrophages [12]. On the basis of CD45 expression, within microglial cell population, CD45^lo^ (resting) and CD45^int^ (activated) cells can be distinguished [41, 42]. Although extremely strongly activated microglia may express CD45 at high levels (CD45^hi^) [42], inflammatory dendritic cells and macrophages are suggested to be CD11b + cells that predominantly display high surface levels of CD45 in spinal cord [12, 40, 42]. It is difficult to discriminate inflammatory dendritic cells from macrophages based on phenotypic markers [40]. In agreement with upregulated expression of GM-CSF in spinal cord mononuclear cells [12], the greater cellularity of CD11b + cell population from aged rats reflected mainly the greater (*p* < 0.001) frequency and number of CD45^hi^ cells, presumably inflammatory dendritic cells/macrophages (Fig. 5). The frequency of CD45^int^ cells among CD11b + cells retrieved from aged rats was less (*p* < 0.001), but their number was greater (*p* < 0.001) compared with young rats (Fig. 5). The boundaries between CD45^int^ and CD45^hi^ expressing populations were settled as previously described [21, 41, 42]. Considering that upregulated CCL2 (monocyte chemoattractant protein-1, MCP-1) expression is important for (i) the accumulation of proinflammatory dendritic cells and macrophages in the CNS during EAE [43, 44] and MS [45], and (ii) chronic EAE development [46, 47], the expression of mRNA for CCL2 was examined. Indeed, the expression of CCL2 mRNAs was upregulated (*p* < 0.001) in aged compared with young rat spinal cord (Fig. 5).

Considering that (i) activation of macrophages [48], dendritic cells [49] and microglial cells [50] is mediated in part by proinflammatory cytokine and TLR signaling pathways that can be negatively regulated by suppressor of cytokine signaling 1 (SOCS1), and (ii) that down-regulation of its expression increases production of proinflammatory cytokines and tissue damaging mediators contributing to the development of chronic inflammatory autoimmune diseases, including MS and EAE [48, 51], SOCS1 expression was also measeured. Indeed, SOCS1 mRNA expression was diminished (*p* < 0.001) in aged compared with young rat spinal cord mononuclear cells (Fig. 5). Consistently, apart from mRNAs for IL-23/p19, IL-1β, the expression of mRNA for TNF-α was also several fold greater (*p* < 0.001) in aged compared with young rat spinal cord mononuclear cells (Fig. 5).

### Aging increases the accumulation of CD11b + CD45^hi^ cells in spinal cord of AO rats on the 7^th^ d.p.i.

**Fig. 6:**
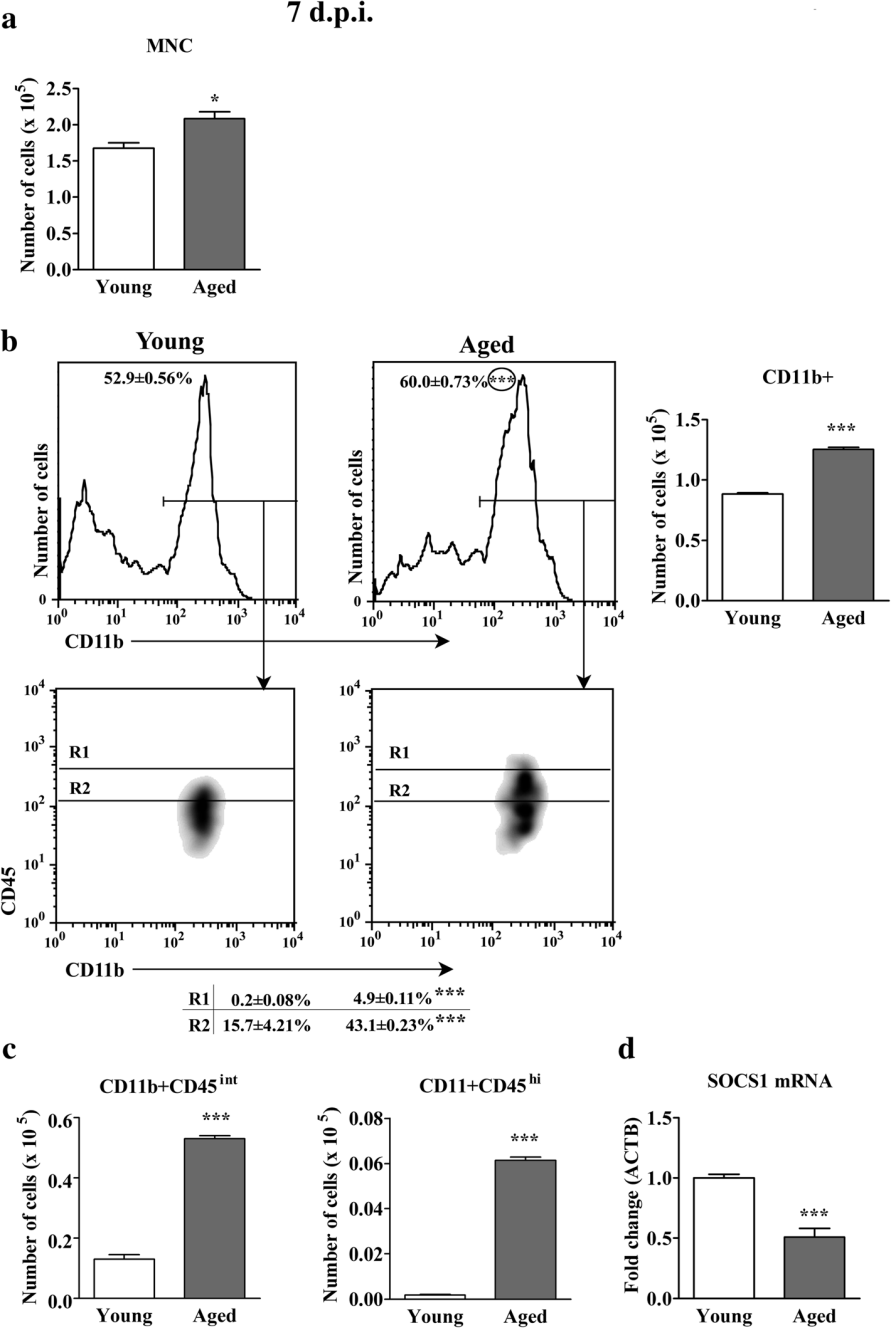
Aging increases the number of all spinal cord mononuclear cells, and CD45^int/hi^ CD11b + nonlymphoid cells, recovered on the 7^th^ d.p.i. from AO rat spinal cord. (**a**) Bar graph indicates the total number of mononuclear cells (MNC) isolated from spinal cord of young and aged rats on the 7^th^ d.p.i. (Panel **b**) Flow cytometry density plots indicate the expression of CD45 on CD11b + cells from spinal cord of (left) young and (right) aged rats on the 7^th^ d.p.i. CD11b + cells were gated as shown in the flow cytometry histograms. Numbers in the flow cytometry histograms represent the percentage of CD11b + cells. R1 = CD11b + CD45^hi^ cells; R2 = CD11b + CD45^int^ cells. Numbers in the table indicate the percentage of cells in the specified region (R). Bar graph indicates the number of CD11b + cells in spinal cords from young and aged rats. (Panel **c**) Bar graphs indicate the number of (left) CD11b + CD45^int^ and (right) CD11b + CD45^hi^ cells in spinal cords from young and aged rats on the 7^th^ d.p.i. (**d**) Bar graph indicates the fold change in expression of mRNA for SOCS1 in spinal cord mononuclear cells from aged relative to young rats on the 7^th^ d.p.i. as determined by RT-qPCR. Data are normalized to β-actin (ACTB). All results are presented as means ± SEM (*n* = 9/group). Data are representative of one of two experiments with similar results. *p < 0.05; ***p < 0.001

Microglial cells become activated in the CNS before the onset of clinical EAE [12]. Their activation is suggested to precede the accumulation of inflammatory dendritic cells and macrophages, which also occurs during the preclinical stage of disease [11]. Thus, to further confirm age-related increase in the accumulation of these cells in spinal cord of AO rats immunized for EAE, on the 7^th^ d.p.i. CD11b + cells were examined for frequency and number, and CD45 expression. Indeed, at this time-point following the immunization, the greater (*p* < 0.05) number of mononuclear cells was retrived from spinal cord of aged compared with young rats, and among them greater (*p* < 0.001) frequency and number of CD11b + cells was detected (Fig. 6). Within CD11b + cell population markedly greater (*p* < 0.001) frequency and number of both CD45^int^ and CD45^hi^ cells was found (Fig. 6). Additionally, although on the 7^th^ d.p.i. the frequency of the spinal cord infiltrating CD4+ TCRαβ + lymphocytes was lower (*p* < 0.05), their number was greater (*p* < 0.05) in aged compared with young rats (Additional file 2: Figure S2).

Furthermore, SOCS1 mRNA expression in spinal cord mononuclear cells was measured. As on the 16^th^ d.p.i., the amount of mRNA for SOCS1 was diminished (*p* < 0.001) in spinal cord mononuclear cells from aged compared with young rats (Fig. 6).

### Immunization for EAE differently affects cellularity and phenotypic profile of draining lymph nodes in aged and young AO rats

Finally, considering that the encephalitogenic CD4+ T cell are primed in draning lymph nodes, and that resistence to EAE in GM-CSF deficient mice is likely to occur due to inefficient T cell priming in the periphery due to an absence of GM-CSF in the lymph node [12], draining lymph node cells were examined for the number of CD4+ TCRαβ + lymphocytes and their expression of activation molecules and IL-17 and GM-CSF.

#### Aging increases the number of activated CD4+ TCRαβ + lymphocytes in draining lymph nodes from rats immunized for EAE

**Fig. 7:**
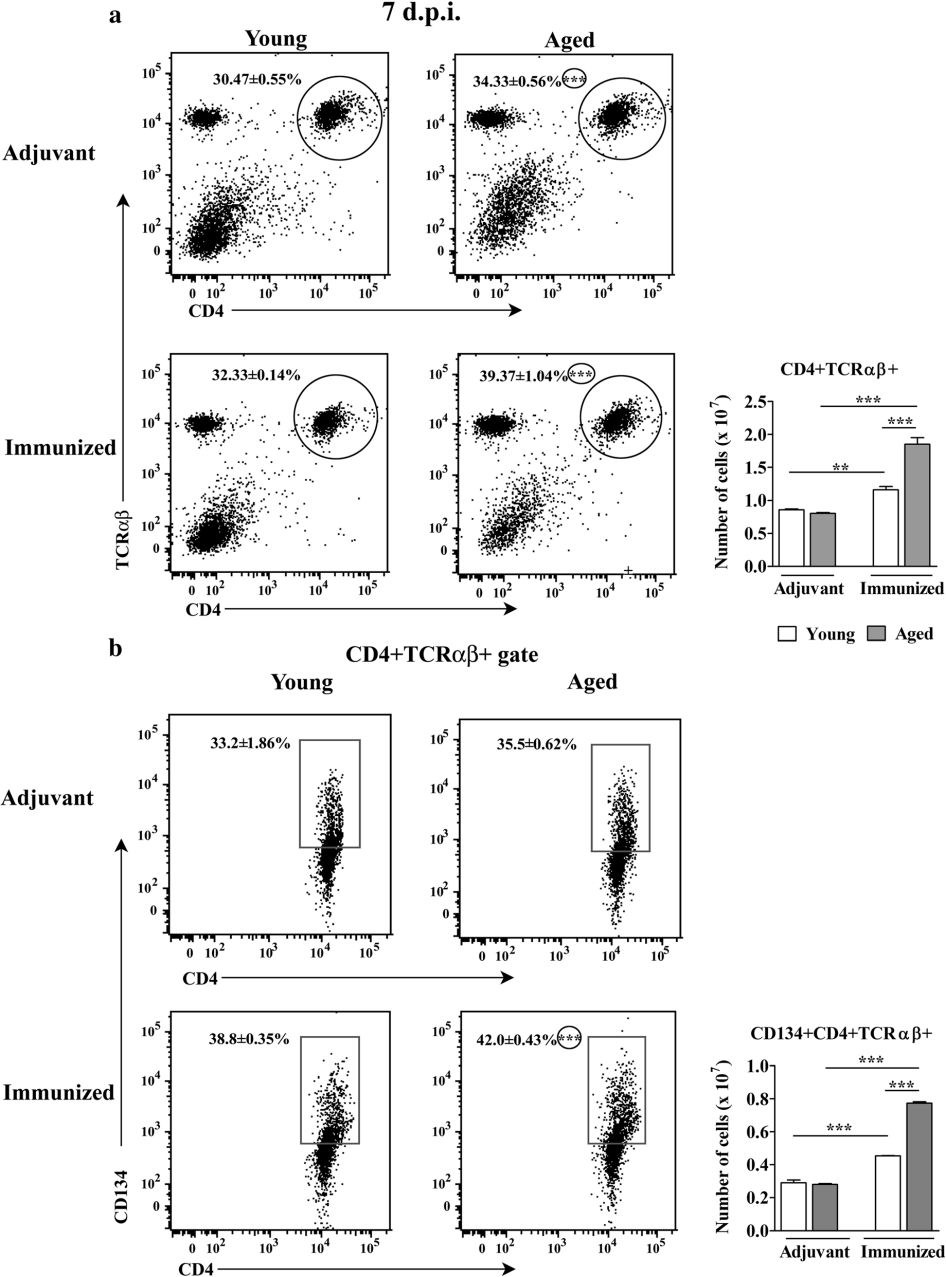
Aging increases the number of CD134 + CD4+ T lymphocytes in draining lymph nodes of AO rats immunized for EAE. (Panel **a**) Flow cytometry dot plots show CD4 vs TCRαβ staining of draining lymph node mononuclear cells from (left) young and (right) aged rats (upper dot plots) injected with CFA and *Bordetella pertussis* (Adjuvant) or (lower dot plots) immunized for EAE (Immunized) on the 7^th^ d.p.i. Bar graph shows the number of CD4+ TCRαβ + cells in draining lymph nodes from young and aged rats injected with adjuvant or immunized for EAE. (Panel **b**) Flow cytometry dot plots show CD134 vs CD4 staining of CD4+ TCRαβ + lymphocytes retrieved from draining lymph nodes of (left) young and (right) aged rats (upper dot plots) injected with CFA and *Bordetella pertussis* (Adjuvant) or (lower dot plots) immunized for EAE on the 7^th^ d.p.i. Bar graph shows the number of CD134 + CD4+ TCRαβ + cells in draining lymph nodes from young and aged rats injected with adjuvant or immunized for EAE. Numbers in the flow cytometry dot plots represent the percentage of cells in the indicated region. All results are presented as means ± SEM (*n* = 9/group). Data are representative of one of two experiments with similar results. **p < 0.01; ***p < 0.001

The lymph node weight and total number of draining lymph node cells were greater (*p* < 0.001) in aged than in young rats immunized for EAE (Additional file 3: Figure S3). To ascertain that these changes were related to immunization with specific (auto)antigen, the same parameters were analyzed in age-matched rats injected with adjuvant, i.e. CFA and *Bordetella pertussis*. There were no age-related differences in the value of any of these two parameters in rats injected with adjuvant (Additional file file 3: Figure S3). This suggested that the age-associated differences in draining lymph node weight and cellularity in rats immunized for EAE were related to the response to autoantigen(s). Furthermore, the frequency of CD4+ TCRαβ + lymphocytes among draining lymph node cells and their number were greater (*p* < 0.001) in aged than in young rats immunized for EAE (Fig. 7). However, only the frequency of CD4+ TCRαβ + lymphocytes was greater (*p* < 0.001) in aged than in young rats injected with adjuvant, but this difference was less (12 % in rats injected with adjuvant vs 23.4 % in rats immunized for EAE) pronounced in these animals than in those immunized for EAE. The number of draining node CD4+ TCRαβ + lymphocytes was comparable between aged and young rats injected with adjuvant (Fig. 7). Thus, age-related difference in the number of CD4+ TCRαβ + lymphocytes in rats immunized for EAE could also be related to the response to autoantigen(s).

Next, CD4+ lymphocytes from rats immunized for EAE were examined for the expression of activation markers. The relative and absolute numbers of CD134+ cells within CD4+ TCRαβ + lymphocytes was greater (*p* < 0.001) in aged compared with young rats (Fig. 7). Given that the values of all of these parameters were comparable between aged and young rats injected with adjuvant (Fig. 7), the age-associated differences observed in their values in rats immunized for EAE could be ascribed to the immunization with autoantigen(s).

#### Aging increases the frequency of IL-17+ cells among CD4+ T lymphocytes in draining lymph nodes from rats immunized for EAE

**Fig. 8:**
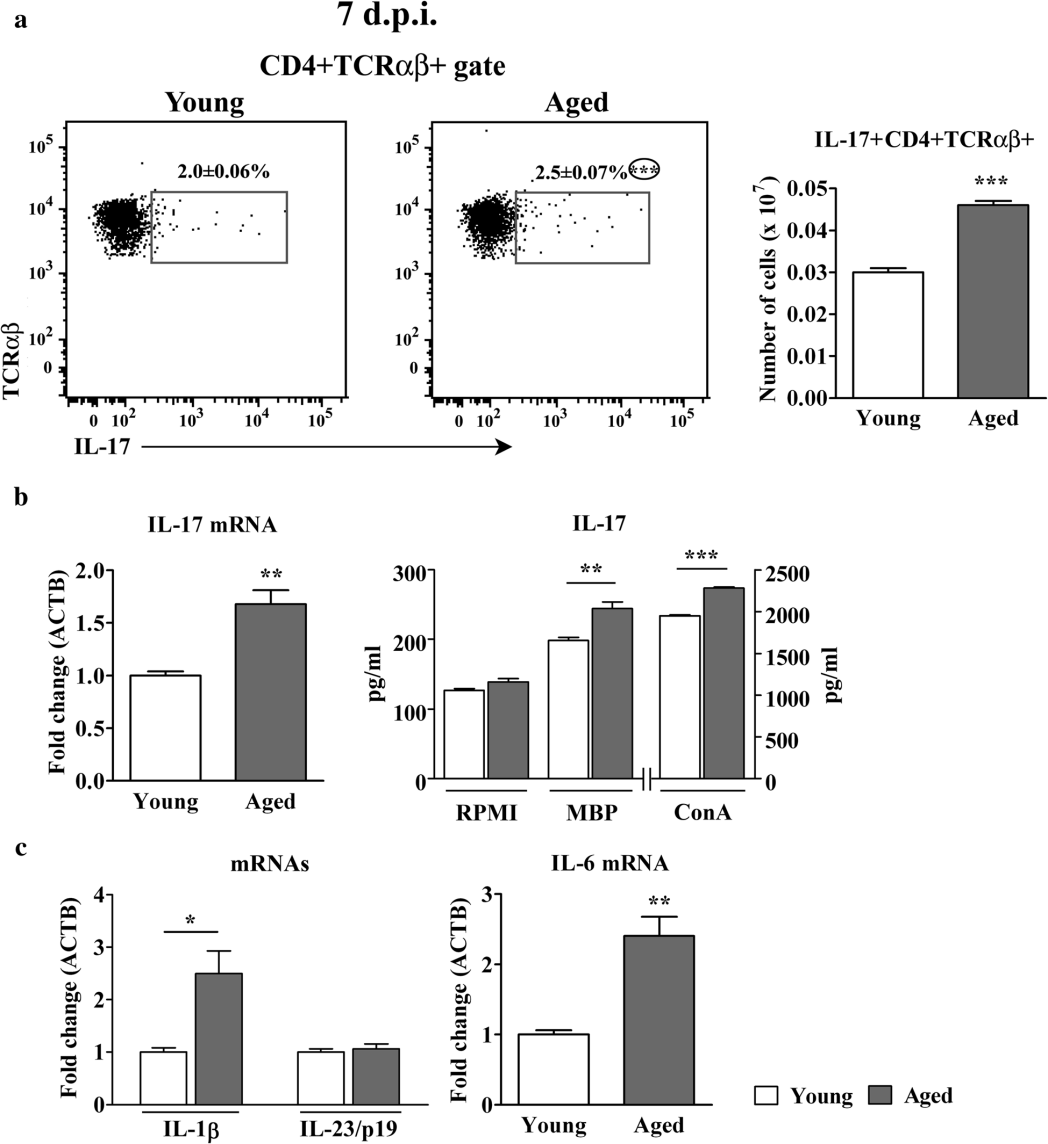
Aging increases the frequency of IL-17+ cells among CD4+ T lymphocytes in draining lymph nodes from AO rats immunized for EAE. (Panel **a**) Flow cytometry dot plots show the expression of IL-17 in CD4+ TCRαβ + lymphocytes retrieved on the 7^th^ d.p.i. from draining lymph nodes of (left) young and (right) aged rats immunized for EAE. Numbers in the flow cytometry dot plots indicate the percentage of cells in the indicated region. Bar graph shows the number of IL-17 + CD4+ TCRαβ + lymphocytes in draining lymph nodes from young and aged rats. (Panel **b**) Left bar graph indicates the fold change in expression of mRNA for IL-17 in CD4+ draining lymph node cells isolated on the 7^th^ d.p.i. using MACS (described in detail in the section Methods) from aged relative to young rats as determined by RT-qPCR. Data are normalized to β-actin (ACTB). Right bar graph shows concentrations of IL-17 in 72 h lymph node mononuclear cell cultures from young and aged rats without ConA or MBP (RPMI) or upon stimulation with either ConA (ConA) or MBP (MBP) as determined by ELISA. (Panel **c**) Bar graphs indicate the fold change in expression of mRNAs for (left) IL-1β and IL-23/p19 and (right) IL-6 in draining lymph node cells on the 7^th^ d.p.i. from aged relative to young rats as determined by RT-qPCR. All results are presented as means ± SEM (*n* = 9/group). Data are representative of one of two experiments with similar results. *p < 0.05; **p < 0.01; ***p < 0.001

The frequency and number of IL-17+ cells within CD4+ TCRαβ + lymphocytes were greater (*p* < 0.001) in aged than in young rats (Fig. 8). Consistently, the expression of IL-17 mRNA was upregulated (*p* < 0.01) in freshly isolated CD4+ cells from draining lymph nodes of aged compared with young rats (Fig. 8). Additionally, in MBP- and ConA-stimulated draining lymph node cell cultures from aged rats the concentration of IL-17 was greater (*p* < 0.01 and *p* < 0.001, respectively) than in the corresponding cultures from young rats (Fig. 8).

Next, considering the previous findings, the expression of the major cytokines driving Th17 cell differentiation, i.e. IL-1β and IL-6, which are required for the induction of IL-17 expression in naive cells, and IL-23, which secures their survival, expansion and pathogenic capacity [8, 9, 52, 53], was examined. The amount of IL-1β (*p* < 0.05) and IL-6 (*p* < 0.01) mRNAs was greater in aged rat draining lymph node cells, whereas that of IL-23/p19 was comparable between aged and young rat draining lymph node cells (Fig. 8).

#### Aging increases the frequency of GM-CSF+ cells among CD4+ T lymphocytes in draining lymph nodes from rats immunized for EAE

**Fig. 9:**
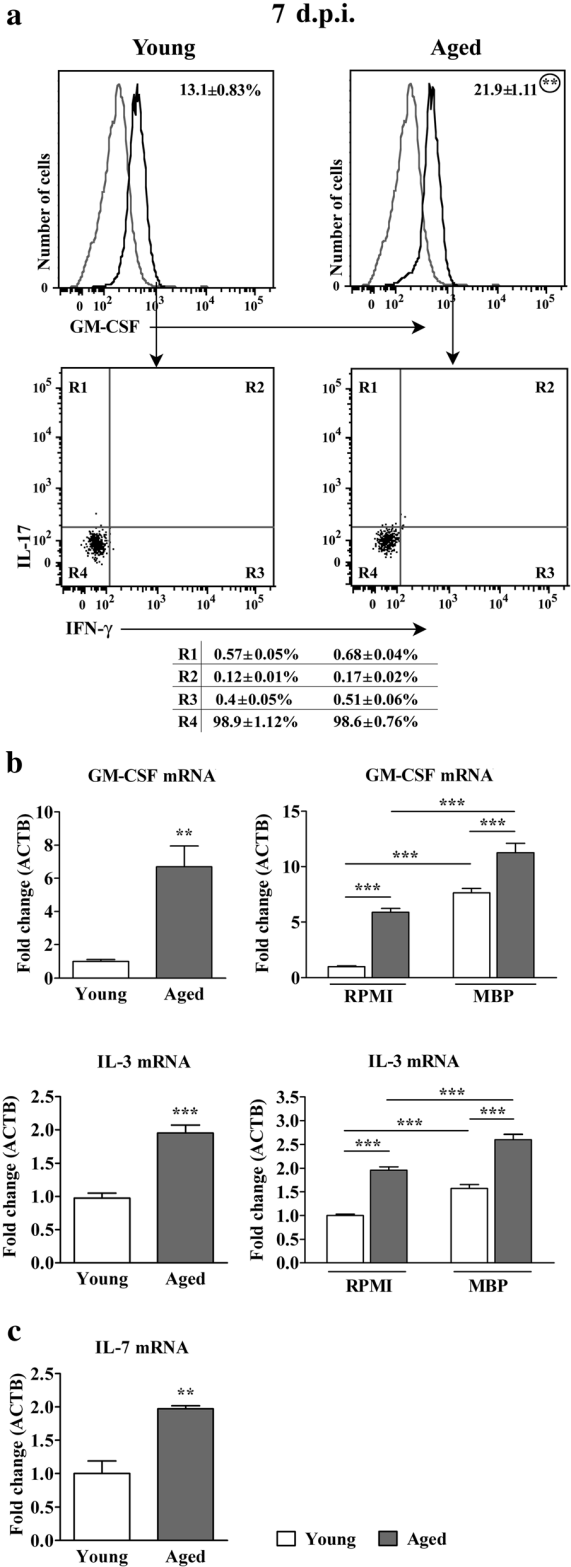
Aging increases the frequency of GM-CSF+ cells among CD4+ T lymphocytes in draining lymph nodes from AO rats immunized for EAE. (Panel **a**) Overlaid Flow cytometry histograms show GM-CSF expression in CD4+ T cells isolated from draining lymph nodes of (left) young and (right) aged rats on the 7^th^ d.p.i. using MACS (described in detail in Methods). In the overlaid flow cytometry histograms left histograms (thin grey line) represent nonspecific binding of secondary antibody. Flow cytometry dot plots show IFN-γ vs IL-17 staining of GM-CSF+ T cells retrieved from draining lymph nodes of (left) young and (right) aged rats on the 7^th^ d.p.i. Numbers in the flow cytometry profiles represent the percentage of cells in the indicated region. (Panel **b**) Left bar graphs show the fold change in expression of mRNAs for (upper) GM-CSF and (lower) IL-3 in CD4+ draining lymph node cells isolated on the 7^th^ d.p.i. using MACS (described in detail in the section Methods) from aged relative to young rats. Right bar graphs show the fold change in expression of mRNAs for (upper) GM-CSF and (lower) IL-3 in draining lymph node cells cultivated for 72 h in RMPI medium from aged rats and MBP-stimulated cells from young and aged rats relative to those from young rats cultivated in RMPI medium, as determined by RT-qPCR. (**c**) Bar graph shows fold change in mRNA expression for IL-7 in draining lymph node tissue on the 7^th^ d.p.i. from aged relative to young rats as determined by RT-qPCR. Data are normalized to β-actin (ACTB). All results are presented as means ± SEM (*n* = 9/group). The data, except for GM-CSF, are representative of one of two experiments with similar results. **p < 0.01; ***p < 0.001

The frequency of GM-CSF+ cells among CD4+ draining lymph node lymphocytes was also higher (*p* < 0.01) in aged than in young rats (Fig. 9). Consistently, the expression of GM-CSF and IL-3 mRNAs was greater (*p* < 0.01 and *p* < 0.001, respectively) in freshly isolated CD4+ cells from aged compared with young rat draining lymph nodes (Fig. 9). In addition, greater (*p* < 0.001) amount of mRNAs for GM-CSF and IL-3 was also found in cells from unstimulated and MBP-stimulated draining lymph node cell cultures from aged rats (Fig. 9).

Next, GM-CSF + CD4+ draining lymph node lymphocytes were examined for IL-17 and IFN-γ production. The majority of these cells did not contain either IL-17 or IFN-γ (Fig. 9). Given that in rats of both ages IL-4 mRNA expression was below the limit of detection, it may be assumed that GM-CSF + IL-17-IFN-γ- cells did not produce IL-4. The frequency of IL-17-IFN-γ- cells among GM-CSF + CD4+ lymphocytes was comparable between aged and young rats (Fig. 9). The relative numbers of cells belonging to other GM-CSF subsets were almost negligiable. Thus, given that frequency of GM-CSF+ cells was higher within CD4+ lymphocytes, it is obvious that the expansion of GM-CSF+ CD4+ subset in aged rats reflected mainly higher frequency of IL-17-IFN-γ- cells (21.59 ± 1.21 % in aged vs 12.96 ± 0.98 % in young rats; *p* < 0.01). Furthemore, as in the spinal cord, the expression of mRNA for IL-7, the cytokine inducing GM-CSF expression in mouse Th-GM lymphocytes [10], was strikingly upregulated (*p* < 0.01) in aged compared with young rat draining lymph node tissue (Fig. 9).

## Discussion

The study showed that resistance of AO rats to EAE diminishes with aging. Differently from young rats, which did not exhibit neurological deficit, majority of aged AO rats developed chronic EAE with mild, but apparent neurological deficit. This is in agreement with data indicating that the age at immunization significantly influences susceptibility to EAE in mice of both sexes [54]. More specifically, it has been shown that in B10.S x SJL/J F(2) intercross mice age effect is capable of overriding eae5 (the H2-linked locus controlling susceptibility to clinical disease)-dependent genetic control of susceptibility to clinical EAE [54]. Furthermore, it should be pointed that the resistance of young adult AO rats to EAE is not absolute, but quantitative, as minimal single cell infiltrate is regularly seen (independently on immunization protocol) in their spinal cord [27]. Additionally, this resistance is suggested not to be due to immune cell inability to recognize determinants of MBP, but rather to the differences in immunoregulatory circuits operating during the induction and/or effector phase of EAE [26, 27]. Consistently, our study revealed several pathogenetically significant differences in both effector and inductive phase of EAE between young and aged AO rats.

In accordance with the differences in clinical outcome of the immunization, on the 16^th^ d.p.i. (when clinical signs of EAE reached the plateau in aged rats) significantly more mononuclear cells was retrieved from aged compared with young rats. In addition, within the isolated cell population from aged rats strikingly more CD11b + cells was identified when compared with young rats. The accumulation of CD45^hi^ cells, presumably mainly inflammatory dendritic cells and macrophages [12, 40], contributed to the expansion of CD11b + population in aged rats. This is consistent with data indicating that blood-borne myeloid cells, such as dendritic cells and macrophages are a prominent constituent of inflammatory infiltrates in the CNS during MS and EAE [55, 56]. Furthermore, blood-borne myeloid cells, i.e. proinflammatory dendritic cells and macrophages, are shown to accumulate in the CNS of transgenic animals that simultaneously express CCL2 and Fms-like tyrosine kinase 3 ligand in the periphery, and these animals spontaneously develop meningeal and perivascular inflammation in association with an ascending paralysis [57]. In other words, the neuroinflammation in this model (i) is primarily driven by myeloid cells and (ii) it is not dependent on either T or B lymphocytes [57]. The enhanced CCL2 expression has been associated with the progression of the CNS pathology in two clinically distinct mouse models of MS, i.e. Theiler's murine encephalomyelitis virus-induced demyelinating disease and relapsing-remitting EAE [58], and the development of relapse of autoimmune encephalomyelitis in Lewis rats [59]. Thus, the upregulated expression of mRNA for CCL2 in aged compared with young rat spinal cord tissue corroborated the increased accumulation of proinflammatory dendritic cells and macrophages in the spinal cord of aged rats. The increased accumulation of proinflammatory dendritic cells, which are thought to be licensed to induce polarization of pathogenic Th17 cells [11], and macrophages in the spinal cord during EAE has been related to the enhanced production of GM-CSF by infiltrating CD4+ T lymphocytes [11, 13]. GM-CSF has been suggested to accelerate the release of bone marrow precursors (so that their number expands more than 60-fold before the onset of EAE) that ultimately differentiate into the CNS-infiltrating dendritic cells and macrophages [11, 13]. Indeed, the expression of GM-CSF mRNA was markedly enhanced in both freshly isolated and in PMA- and ionomycin-stimulated spinal cord mononuclear cells. Considering that IFN-γ production by CD4+ T cells was dramatically increased following priming by GM-CSF-treated microglia [35], the augmented expression of GM-CSF may also explain the upregulated expression of IFN-γ mRNA in aged rat spinal cord mononuclear cells despite the comparable frequency of IFN-γ + cells within CD4+ T lymphocytes infiltrating aged and young rat spinal cord. The greater expression of GM-CSF mRNA in aged rat spinal cord mononuclear cells was consistent with higher frequency of GM-CSF+ cells within CD4+ T lymphocytes infiltrating the spinal cord of aged rats compared with young ones. Moreover, within spinal cord infiltrating GM-CSF+ CD4+ T lymphocytes from aged rats the frequency of IL-17 + IFN-γ + cells was greater when compared with young rats. Unlike in humans, in whom GM-CSF is not a specific cytokine of Th17 cells given its disconnection from IL-17 expression, its production by RORγt- Th cells, and its down-regulation by Th17 cell-priming cytokines [37], GM-CSF + IL-17 + IFN-γ + CD4+ T lymphocytes are suggested to be highly pathogenic in mice [8, 9].

In addition, within CD4+ T lymphocytes infiltrating spinal cord of aged rats the higher frequency of GM-CSF + IL-17-IFN-γ- cells was found. These cells most likely did not produce IL-4 in either young or aged rats. The previous findings, in conjunction with the greater expression of mRNA for IL-3, the cytokine showing the same production pattern as GM-CSF in mouse CD4+ T cells [10], in fresh and PMA- and ionomycin-stimulated aged rat mononuclear spinal cord cells, and IL-7, the cytokine driving GM-CSF expression in mouse Th-GM lymphocytes [10], in aged rat spinal cord tissue, suggest that the greater accumulation of GM-CSF+ CD4+ lymphocytes in spinal cord also contributed to more severe clinical outcome of the immunization in aged compared with young rats. It favor of the previous assumption are data indicating that dysregulation of the IL-7/IL-7R axis has long been implicated in autoimmune diseases, such as type 1 diabetes, multiple sclerosis and rheumatoid arthritis [60, 61].

Furthermore, the greater number of the spinal cord infiltrating CD4+ TCRαβ + lymphocytes and greater frequency and number of CD134+ reactivated cells among them was consistent with the more severe clinical outcome of the immunization in aged compared with young rats.

We did not identify the bone marrow precursors that ultimately differentiate into CNS-infiltrating dendritic cells and macrophages in blood, but we showed that the frequency of CD11b + cells expressing morphological characteristics of monocytes/macrophages according to FSC and SSC parameters was increased in blood from aged rats immunized for EAE, but not in these rats injected with adjuvant (Additional file 4: Figure S4). Thus, it may be speculated that the increase in their frequency in aged rats immunized for EAE was related to the immunization with autoantigen(s). This was consistent with age-associated increases in number of CD14 + CD16+ inflammatory monocytes, which has been shown in humans [62, 63]. The accumulation of inflammatory dendritic cells and macrophages is shown to occur during the preclinical stage of disease [11]. Indeed on the 7^th^ d.p.i. significantly greater proportion and number of CD11b + cells was found within mononuclear cells retrieved from aged compared with young rat spinal cord. Differently from young rat CD11b + cells, which encompassed negligible number of CD45^hi^ cells indicating a lack of spinal cord infiltration with inflammatory dendritic cells and macrophages [12], a significant frequency of these cells was registered among CD11b + cells from aged rats. Consequently, on the 7^th^ d.p.i., in addition to activated microglial CD45^int^ cell number, the number of CD45^hi^ cells, presumably mainly inflammatory dendritic cells and macrophages [12, 40], was strikingly greater in aged than in young rat spinal cord.

Next, considering that the downregultion of SOCS1 expression is shown to be involved in the development of MS and EAE [48, 51], the expression of mRNA for this cytokine in spinal cord mononuclear cells was also measured. Indeed, SOCS1 mRNA expression was less in spinal cord mononuclear cells from aged compared with young rats. Consistently, on the 16^th^ d.p.i. the expression of proinflammatory cytokines IL-1β, IL-23/p19 and TNF-α was markedly upregulated in spinal cord mononuclear cells from aged compared with young rats. Thus, it seems likely that the expansion of CD45^int^ and CD45^hi^ subpopulations from aged rats was followed by enhanced expression of proinflammatory cytokines in these cells due to dysregulation of their SOCS1 expression.

Given that resistence to EAE in GM-CSF deficient mice is suggested to occur due to inefficient T- cell priming in the draining lymph node to the myelin oligodendrocyte glycoprotein [12], draining lymph node cells were examined for the presence of activated CD4+ T lymphocytes and the expression of GM-CSF. In agreement with greater number of the spinal cord infiltrating CD4+ TCRαβ + lymphocytes on the 7^th^ and 16^th^ d.p.i., the greater number of activated CD4+ TCRαβ + lymphocytes was found in draining lymph nodes from aged compared with young rats. The analyses of CD4+ TCRαβ + lymphocyte profile in age-matched rats immunized with CFA only showed that this difference was specific to immunization with specific (auto)antigens. This was consistent with data indicating the accumulation of T cells with autoreactive specificities with aging in both humans and experimental animals [20, 64]. This phenomenon has been related to the erosion of naïve T cell pool with aging, and consequent residual T-cell proliferation to reconstitute their nearly normal numbers [65]. In this compensatory event T cells with higher avidity for self-peptide/MHC complexes enjoy advantage and expand more relative to the low avidity T cells [20]. This leads to the accumulation of high avidity T cells, which is potentially dangerous due to increased risk of autoimmune disorders [66]. The lack of increase in the incidence of the most autoimmune diseases with aging has been related to the expansion of many protective regulatory mechanisms [64].

Furthermore, the greater expression of GM-CSF in draining lymph node cells from aged rats following MBP stimulation *in vitro* suggested enhanced production of GM-CSF by encephalitogenic CD4+ draining lymph node T lymphocytes of aged compared with young rats. FCA showed that GM-CSF+ CD4+ lymphocytes in draining lymph nodes from rats of both ages exhibited predominantly IL-17-IFN-γ- phenotype, whereas the frequencies of cells belonging to other GM-CSF subsets were almost negligible. The frequency of GM-CSF + IL-17-IFN-γ- cells was greater within CD4+ lymphocytes from aged rat draining lymph nodes. Consistently, IL-3 mRNA in fresh CD4+ draining lymph node lymphocytes and IL-7 mRNA expression in draining lymph node tissue was greater in aged than in young rats. In addition, the higher frequency of IL-17+ cells among CD4+ draining lymph node lymphocytes from aged rats was found. The expression profile of Th polarization driving cytokines [52, 53] corroborated this finding. Consistently, upon stimulation with MBP draining lymph node cells from aged rats produced more IL-17 compared with the corresponding cells from young rats. At the first glance, our findings indicating an extremely limited frequency of GM-CSF + IL-17 + IFN-γ + cells in draining lymph nodes of aged rats were at odds with the observed frequency of these cells in their spinal cord on the 16^th^ d.p.i. However, it should be pointed out that in mice the expression of IL-23/p19, the crucial cytokine for GM-CSF + IL-17 + IFN-γ + cells generation [8], in the CNS occurs only after the onset of EAE [10]. Therefore, it is assumed that: i) IL-23 is not required for the initiation of EAE and ii) GM-CSF-producing Th-GM cells induce the expression of IL-23 from dendritic cells, macrophages, and other CNS-residential cells to sustain the inflammation, i.e. that pathogenic Th17 cells (co-producing IFN-γ and GM-CSF) arise in the CNS to cooperate with Th-GM subset in EAE development [10].

## Conclusions

In conclusion, the study supports the notion that aging may overcome genetic resistance to EAE [54], and points to mechanisms standing behind this phenomenon in AO rats. More specifically, it indicates that enhanced generation of encephalitogenic GM-CSF + IL-17-IFN-γ- CD4+ T lymphocytes in draining lymph nodes and their enhanced infiltration in the spinal cord leads to upregulation of IL-23 in innate immune cells from aged rat spinal cord. Consequently, the generation of pathogenic GM-CSF + IL-17 + IFN-γ + CD4+ T lymphocytes increases in spinal cord, and they, together with accumulated proinflammatory blood-borne myeloid cells, secure sustained neuroinflammation leading to the development of mild chronic EAE in aged AO rats.

## Methods

### Experimental animals

Female young (2-3-month-old) and aged (24-26-month-old) AO rats obtained from a breeding colony in the animal facility of the Immunology Research Centre “Branislav Janković” in Belgrade were used in the present study. The animals were maintained under a 12-h light/dark cycle in a temperature-controlled environment and were provided with standard laboratory food and tap water *ad libitum*. All experimental procedures and animal care were performed in accordance with the Directive 2010/63/EU of the European Parliament and of the Council on the protection of animals used for scientific purposes (revising Directive 86/609/EEC) and approved by the Institutional Animal Care and Use Committee. In the preliminary experiment, 13 of 22 rats were followed over a 60-day-long follow-up period. Differently from young rats, which did not develop clinical EAE, a significant percentage (63 %) of aged rats exhibited neurological deficit. This deficit reached plateau on the 16^h^ d.p.i. in aged rats. For analyses rats (9 per group) were sacrificed on the 16^th^ d.p.i. In the subsequent experiments, on the 16^th^ d.p.i. the spinal cord tissue was collected from rats of both ages for: i) cytokine/chemokine quantification using RT-qPCR and ii) isolation of mononuclear cells for FCA and proinflammatory mediator and/or SOCS1 expression measurement using RT-qPCR. Besides, on the 7^th^ d.p.i. spinal cords and draining lymph nodes were isolated. The mononuclear cells were subjected to FCA of surface and/or intracellular antigen expression and/or SOCS1/cytokine RT-qPCR and/or ELISA quantification. To provide reasonable number of spinal cord mononuclear cells for analyses, spinal cord mononuclear cells were pooled (3 spinal cords/pool).

### Induction and clinical evaluation of EAE

EAE was induced by an intradermal injection of 100 μl of an emulsion made of equal volumes of rat spinal cord homogenate in phosphate-buffered saline (PBS) and CFA containing 1 mg/ml of heat-killed and dried *Mycobacterium tuberculosis* H37Ra (Sigma-Aldrich Chemie GmbH, Taufkirchen, Germany) in the left hind paw, followed by a subcutaneous injection of 0.25 ml of saline suspension of 5x10^8^*Bordetella pertussis* (obtained from Institute of Virology, Vaccines and Sera "Torlak", Belgrade, Serbia) on the dorsum of the same paw. Rats were weighed and graded daily for neurological signs by two independent experienced observers, as follows: 0, no clinical signs; 0.5, distal tail atony; 1, complete tail atony; 2, paraparesis; 3, paraplegia; 4, tetraplegia, moribund state or death.

### Antibodies and immunoconjugates

For immunolabeling, the following mAbs were used: phycoerythrin (PE)-conjugated anti-CD4 (clone OX-38), biotin-conjugated anti-CD8 (clone OX-8), biotin-conjugated anti-CD134 (clone OX-40), biotin-conjugated anti-CD45 (clone OX-1), fluorescein isothiocyanate (FITC)-conjugated anti-IFN-γ (clone DB-1), PE-conjugated anti-IL17A (clone TC11-18H10), as well as peridinin-chlorophyll-protein (PerCP)-conjugated streptavidin and isotype controls, all obtained from BD Biosciences Pharmingen (Mountain View, CA, USA). FITC-conjugated anti-CD11b (clone ED8) mAb was supplied by Serotec (Oxford, UK). Alexa Fluor 647-conjugated TCRαβ (clone R73) and PerCP/cyanine (Cy) 5.5-conjugated goat-anti mouse IgG were provided by BioLegend (San Diego, CA, USA), while purified anti-GM-CSF mAb (clone 83308) was purchased from R&D Systems, Inc. (Minneapolis, MN, USA).

### Isolation of mononuclear cells

For mononuclear cell isolation, rats were deeply anesthetized by an i.p. injection of 80 mg/kg: 8 mg/kg of body weight of ketamine: xylazine anesthetic solution (ketamine, 100 mg/ml Ketamidor, Richter Pharma AG, Wels, Austria; xylasine, 20 mg/ml Xylased, Bioveta, Ivanovice na Hané, Czech Republic and saline, mixed in a 1:0.5:8.5 ratio) and perfused with PBS. Following perfusion, spinal cords and lymph nodes were removed, weighed and grinded on 70 μm nylon cell strainer (BD Biosciences, Erembodegem, Belgium) placed in a petri dish containing ice-cold phosphate buffered saline (PBS) supplemented with 2 % fetal calf serum (FCS, Gibco, Grand Island, NY, USA) and 0.01 % NaN_3_ (Sigma-Aldrich Chemie GmbH) (FACS buffer) or RPMI 1640 medium (Sigma-Aldrich Chemie GmbH) with 5 % FCS (spinal cord cells). Thereby obtained spinal cord single-cell suspensions were further fractioned on a discontinuous 40/70 % Percoll (Sigma-Aldrich Chemie GmbH) gradient at 1000x*g* for 50 min. Spinal cord mononuclear cells from the interface were collected and, as lymph node cells, counted in 0.2 % trypan blue solution using an improved Neubauer hemacytometer.

### Analyses of cytokine intracellular staining and/or expression in spinal cord and draining lymph node mononuclear cells

#### Stimulation of spinal cord and lymph node mononuclear cells for analyses of cytokine intracellular staining

Freshly isolated and MACS-sorted CD4+ spinal cord and draining lymph node cells were cultured in 24-well plates (Sarstedt AG & Co., Nümbrecht, Germany) in 500 μl culture medium supplemented with 200 ng/ml phorbol 12-myristate 13-acetate (PMA, Sigma-Aldrich Chemie GmbH) and 400 ng/ml ionomycin (Sigma-Aldrich Chemie GmbH). The culture medium consisted of RPMI 1640 supplemented with 2 mM L-glutamine (Serva, Heidelberg, Germany), 1 mM sodium pyruvate (Serva), 100 units/ml penicillin (ICN, Costa Mesa, CA, USA), 100 μg/ml streptomycin (ICN) and 10 % FCS. The plates were incubated in a humidified atmosphere of 5 % v/v CO_2_ for 4 h at 37 °C. For intracellular staining of cytokines 3 μg/ml of brefeldin A (eBioscience) was added 2 h before the end of the assay. Following incubation, the cells were harvested for immunostaining and RT-qPCR cytokine quantification.

#### Cultivation of draining lymph node cells for analyses of T-cell cytokine production

All draining lymph node cell cultures were run in culture medium in a 5 % CO_2_ humidified air atmosphere. For analysis of cytokine production, draining lymph node cells were cultured for 72 h in the culture medium alone or in culture medium supplemented with 20 μg/ml MBP (Sigma-Aldrich Chemie GmbH) or 2.5 μg/ml ConA (Sigma-Aldrich Chemie GmbH). Cells were collected for RT-qPCR, whereas cell-free supernatants were harvested and assayed for IL-17 by ELISA.

### Isolation of CD4+ cells from draining lymph node cell suspensions

To examine cytokine expression in CD4+ lymphocytes, mononuclear cells from draining lymph node were subjected to cell separation using MACS. In brief, draining lymph node cell suspensions were incubated with rat CD4 microbeads (clone OX-38, Miltenyi Biotec, Gladbach, Germany) for 15 min at 4 °C. After washing in MACS buffer (degassed PBS with 0.5 % bovine serum albumin and 2 mM ethylenediaminetetraacetic acid), the cells were loaded onto LS column (Miltenyi Biotec) and placed in the magnetic field of the Quadro MACS separator (Miltenyi Biotec) for positive selection. CD4+ spinal cord T cells were purified using a two-step procedure. Firstly, the spinal cord mononuclear cells were labeled using rat CD8a microbeads (clone G28, Miltenyi Biotec) and passed through a magnetic field for negative selection. In the second step, CD8- cells were incubated with rat pan T-cell microbeads (clone OX-52, Miltenyi Biotec) and positively selected for T lymphocytes, as described above. FCA revealed that positive fractions contained 90–95 % of CD4+ cells.

### RT-qPCR quantification of cytokine, chemokine and SOCS1 mRNAs

Spinal cord and draining lymph node cells and/or tissue samples were harvested using Nucleic Acid Purification Lysis Solution (Applied Biosystems, Foster City, CA, USA) and immediately stored at -70 °C until RNA purification. Total RNA from either 1 × 10^5^ lysed cells or spinal cord/lymph node tissue homogenates was extracted using ABI Prism 6100 Nucleic Acid PrepStation system (Applied Biosystems) and Total RNA Chemistry Starter Kit (Applied Biosystems). The procedure included DNAse treatment to ensure that no genomic DNA contamination was present. Isolated RNA was converted to cDNA using High Capacity cDNA Reverse Transcription Kit (Applied Biosystems), in 20-μl reactions ran under optimal conditions (10 min, 25 °C; 120 min, 37 °C; 5 s, 85 °C).

For the analysis of cytokine and enzyme expression, triplicate 25-μl RT-qPCR reactions, containing 1x TaqMan Gene Expression Master Mix (Applied Biosystems), 1x mix of premade primer and hydrolysis probe sets (TaqMan Gene Expression Assays, Applied Biosystems) and 5 μl of cDNA template, were ran under the default Applied Biosystems 7500 Real-Time PCR System conditions (2 min at 50 °C, 10 min at 95 °C, followed by 40 cycles consisted of 15 s at 95 °C and 1 min at 60 °C incubations each).

All the procedures were performed according to the manufacturers’ instructions. Predesigned TaqMan Gene Expression Assays (Applied Biosystems) used in the study are listed in Table 1. Relative differences in target mRNA levels were assessed by the 2^-ΔΔCt^ method with SDS v1.4.0. software (Applied Biosystems), using β-actin as a normalizer, as it has been suggested [67].

**Table 1:** Summary of mRNA targets and reference gene for RT-qPCR analysis

	Symbol	Gene name	Accession No.^**a**^	Assay ID
1	Il1b	Interleukin 1 beta	NM_031512.2	Rn99999009_m1
2	Il3	Interleukin 3	NM_031513.1	Rn00580435_m1
3	Il4	Interleukin 4	NM_201270.1	Rn99999010_m1
4	Il6	Interleukin 6	NM_012589.1	Rn99999011_m1
5	Il7	Interleukin 7	NM_013110.2	Rn00681900_m1
6	Il17a	Interleukin 17A	NM_001106897.1	Rn01757168_m1
7	Il23a	Interleukin 23, alpha subunit p19	NM_130410.2	Rn00590334_g1
8	Ifng	Interferon gamma	NM_138880.2	Rn00594078_m1
9	Csf2	Colony stimulating factor 2 (granulocyte-macrophage)	NM_053852.1	Rn01456850_m1
10	Tnf	Tumor necrosis factor	NM_012675.3	Rn99999017_m1
11	Ccl2	Chemokine (C-C motif) ligand 2	NM_031530.1	Rn00580555_m1
12	Socs1	Suppressor of cytokine signaling 1	NM_145879.2	Rn00595838_s1
13	Actb	Actin, beta	NM_031144.2	Rn00667869_m1

^**a**^RefSeq: NCBI Reference Sequence Database

### FCA

Before immunostaining, mononuclear cells isolated from rat spinal cords and draining lymph nodes were extensively washed in FACS buffer. All incubation steps were performed in the dark on ice. The stained samples were acquired on FACSCalibur or FACSVerse flow cytometer (Becton Dickinson, Mountain View, CA, USA), using CELLQuest or FACSuite v1.0.5.3841 software (Becton Dickinson). FlowJo software version 7.8. (TreeStar Inc, Ashland, OR, USA) was used to analyze the data for percentage of a marker-positive cells. Dead cells and debris were excluded from the analyses by selective gating based on forward scatter (FSC) and side scatter (SSC), whereby background staining level for each fluorochrome type was defined using non-specific IgG isotype-matched controls. Fluorescence minus one (FMO) controls were applied to settle gating boundaries [21].

#### Surface antigen immunostaining

Cells were incubated with saturating concentrations of either fluorochrome-labeled (direct fluorescence) or biotin-conjugated (indirect fluorescence) mAbs for 30 min and washed. When biotin-conjugated mAbs were applied, cells were incubated with PerCP-conjugated streptavidin for additional 30 min and then a final washing step was undertaken before re-suspending the cells in PBS. To determine cell apoptosis, after labeling with PE-conjugated anti-CD4 and Alexa Fluor 647-conjugated anti-TCRαβ mAb cocktail, spinal cord cells were washed with PBS and then with 1 X Annexin V binding buffer (BD Pharmingen). After washing, the cells were incubated with 5 μl Annexin V-FITC (BD Pharmingen) for 15 min at room temperature in the dark and collected for FCA.

#### Intracellular cytokine staining

Spinal cord and lymph node mononuclear cells stimulated with PMA and ionomycin were stained for surface TCRαβ and CD8 antigens and then washed and fixed/permeabilized overnight at 4 °C, using the solutions from fixation/permeabilization buffer kit (eBioscience; http://www.ebioscience.com/resources/best-protocols/flow-cytometry-protocols.htm). Next, for double IFN-γ/IL-17 staining, the cells were washed using appropriate permeabilization buffer (eBioscience), incubated with fluorochrome-conjugated anti-IFN-γ and anti-IL-17 mAbs for 30 min at room temperature in the dark, and then washed again and collected for FCA. For GM-CSF analyses, the fixed MACS-sorted CD4+ cells were sequentially labeled using anti-GM-CSF mAb, PerCP/Cy5.5-conjugated goat anti-mouse IgG and fluorochrome-conjugated anti-IFN-γ/anti-IL-17 mAb cocktail, as described above.

### ELISA

For measuring the IL-17 concentration commercial kit (BioLegend) was used. The assay was performed following the manufacturer’s instructions. A standard curve was generated with the limit of detection for IL-17 of 8 pg/ml.

### Statistical analysis

The statistical significance of differences between groups was assessed using One-way ANOVA followed by Tukey test for post hoc comparisons or Student's unpaired *t*-test. All statistical analyses were performed using GraphPad Prism 5 software (GraphPad Software, Inc., La Jolla, CA, USA). Values of *p* ≤ 0.05 were considered significant.

## Additional files

Additional file 1: Figure S1. Aging does not change the percentage of apoptotic cells among CD4+ TCRαβ + cells in spinal cord of AO rats immunized for EAE. Flow cytometry dot plots show Annexin V vs CD4 staining of T lymphocytes retrieved from spinal cords of (left) young and (right) aged rats on the 16^th^ d.p.i. Numbers in the flow cytometry dot plots represent the percentage of Annexin V+ cells among CD4+ TCRαβ + lymphocytes. Results are presented as means ± SEM (n = 9/group). Data are representative of one of two experiments with similar results. (TIFF 228 kb) Additional file 2: Figure S2. Aging increases the number of spinal cord CD4+ TCRαβ + cells on the 7^th^ d.p.i. in AO rats immunized for EAE. Flow cytometry dot plots show TCRαβ vs CD4 staining of lymphocytes retrieved from spinal cords of (left) young and (right) aged rats on the 7^th^ d.p.i. Numbers in the flow cytometry dot plots represent the percentage of CD4+ TCRαβ + lymphocytes. Bar graph shows the number of CD4+ TCRαβ + cells in young and aged rat spinal cords. Results are presented as means ± SEM (*n* = 9/group). Data are representative of one of two experiments with similar results. **p* < 0.05. (TIFF 562 kb) Additional file 3: Figure S3. Aging increases weight and cellularity of draining lymph nodes from AO rats immunized for EAE. Bar graphs indicate the (left) lymph node (LN) weight and (right) number of draining lymph node mononuclear cells (MNC) from young and aged rats injected with CFA and *Bordetella pertussis* (Adjuvant) or immunized for EAE (Immunized). All results are presented as means ± SEM (*n* = 9/group). Data are representative of one of two experiments with similar results. **p* < 0.05; ****p* < 0.001. (TIFF 168 kb) Additional file 4: Figure S4. Aging increases the frequency of CD11b + cells with monocyte/macrophage physical characteristics in peripheral blood from rats immunized for EAE. (Panel A) Flow cytometry dot plots indicate peripheral blood CD11b + monocyte/macrophage gating strategy. Flow cytometry dot plot indicates (right) CD11b expression on monocytes/macrophages gated according to FSC and SSC as shown in (left) flow cytometry dot plot. (B) Bar graph shows percentage of CD11b + monocytes/macrophages within peripheral blood cells from young and aged rats injected with CFA and *Bordetella pertussis* (Adjuvant) or immunized for EAE (Immunized). All results are presented as means ± SEM (*n* = 9/group). Data are representative of one of two experiments with similar results. ****p* < 0.001. (TIFF 1294 kb)

## Abbreviations

MS: Multiple sclerosis
CNS: Central nervous system
EAE: Experimental autoimmune encephalomyelitis
GM-CSF: Granulocyte macrophage colony-stimulating factor
AO: Albino Oxford
MBP: Myelin basic protein
MCP-1: Monocyte chemoattractant protein-1
SOCS1: Suppressor of cytokine signaling 1
CFA: Complete Freund's adjuvant
PE: Phycoerythrin
FITC: Fluorescein isothiocyanate
PerCP: Peridinin-chlorophyll-protein
FSC: Forward scatter
SSC: Side scatter
FCA: Flow cytometry analysis
PMA: Phorbol 12-myristate 13-acetate
