# Supplementary figures and images for: Aging diminishes the resistance of AO rats to EAE: putative role of enhanced generation of GM-CSF Expressing CD4+ T cells in aged rats
Source: Immun Ageing. 2015 Oct 6;12:16. doi: 10.1186/s12979-015-0044-x (PMC4596406; doi:10.1186/s12979-015-0044-x)

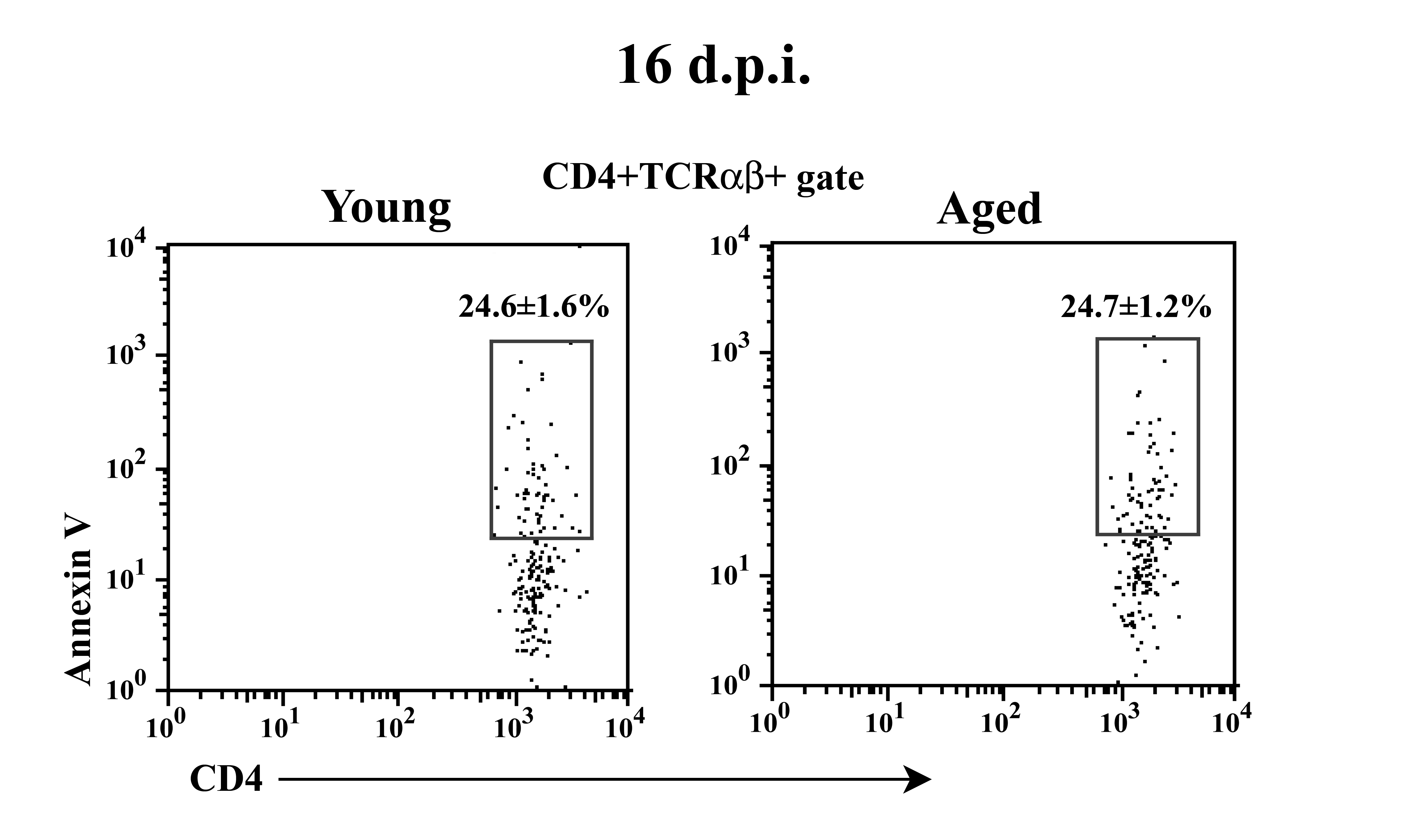

Supplement: Additional file 1: Figure S1. — Aging does not change the percentage of apoptotic cells among CD4+ TCRαβ + cells in spinal cord of AO rats immunized for EAE. Flow cytometry dot plots show Annexin V vs CD4 staining of T lymphocytes retrieved from spinal cords of (left) young and (right) aged rats on the 16th d.p.i. Numbers in the flow cytometry dot plots represent the percentage of Annexin V+ cells among CD4+ TCRαβ + lymphocytes. Results are presented as means ± SEM (n = 9/group). Data are representative of one of two experiments with similar results. (TIFF 228 kb) [file 12979_2015_44_MOESM1_ESM.tif]

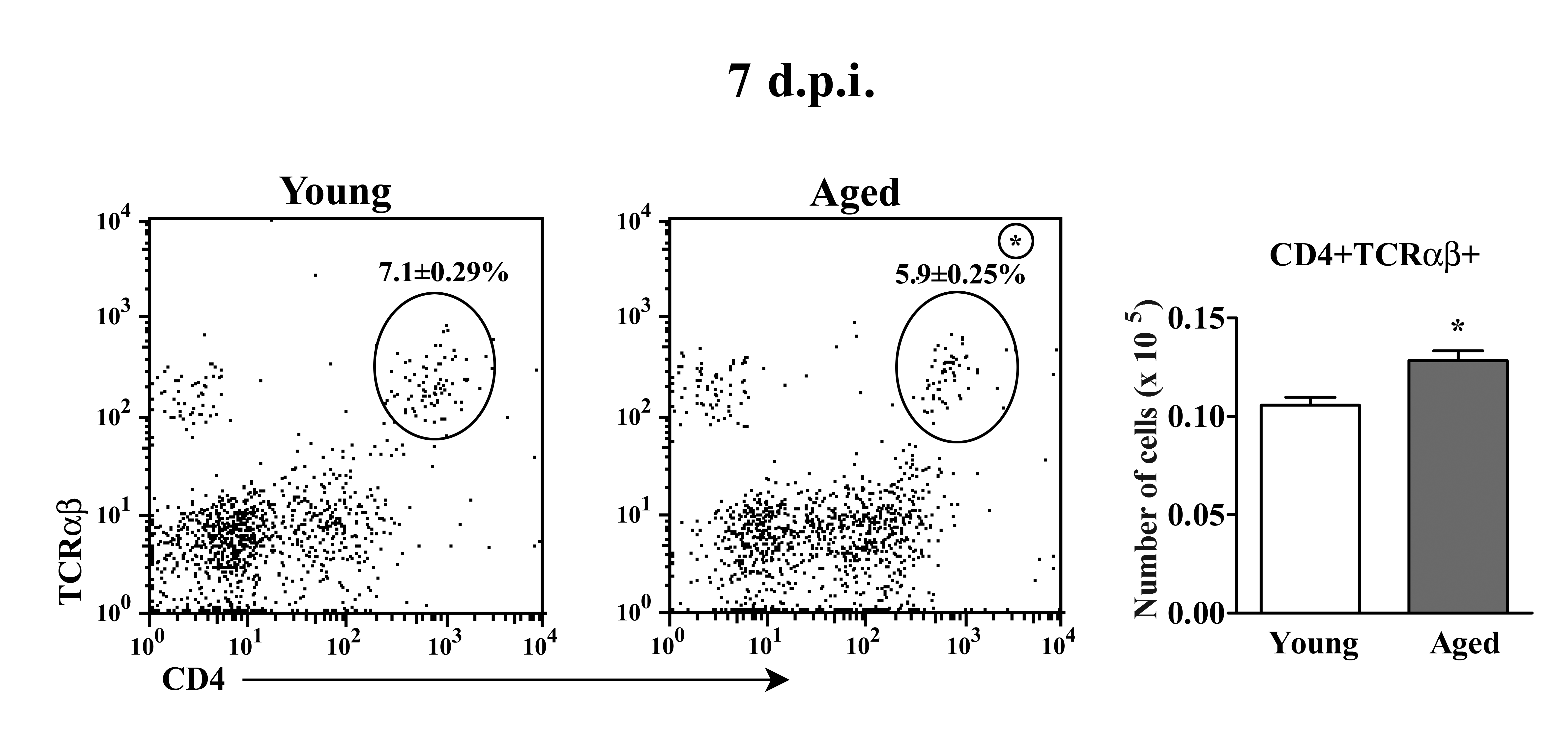

Supplement: Additional file 2: Figure S2. — Aging increases the number of spinal cord CD4+ TCRαβ + cells on the 7th d.p.i. in AO rats immunized for EAE. Flow cytometry dot plots show TCRαβ vs CD4 staining of lymphocytes retrieved from spinal cords of (left) young and (right) aged rats on the 7th d.p.i. Numbers in the flow cytometry dot plots represent the percentage of CD4+ TCRαβ + lymphocytes. Bar graph shows the number of CD4+ TCRαβ + cells in young and aged rat spinal cords. Results are presented as means ± SEM (n = 9/group). Data are representative of one of two experiments with similar results. *p < 0.05. (TIFF 562 kb) [file 12979_2015_44_MOESM2_ESM.tif]

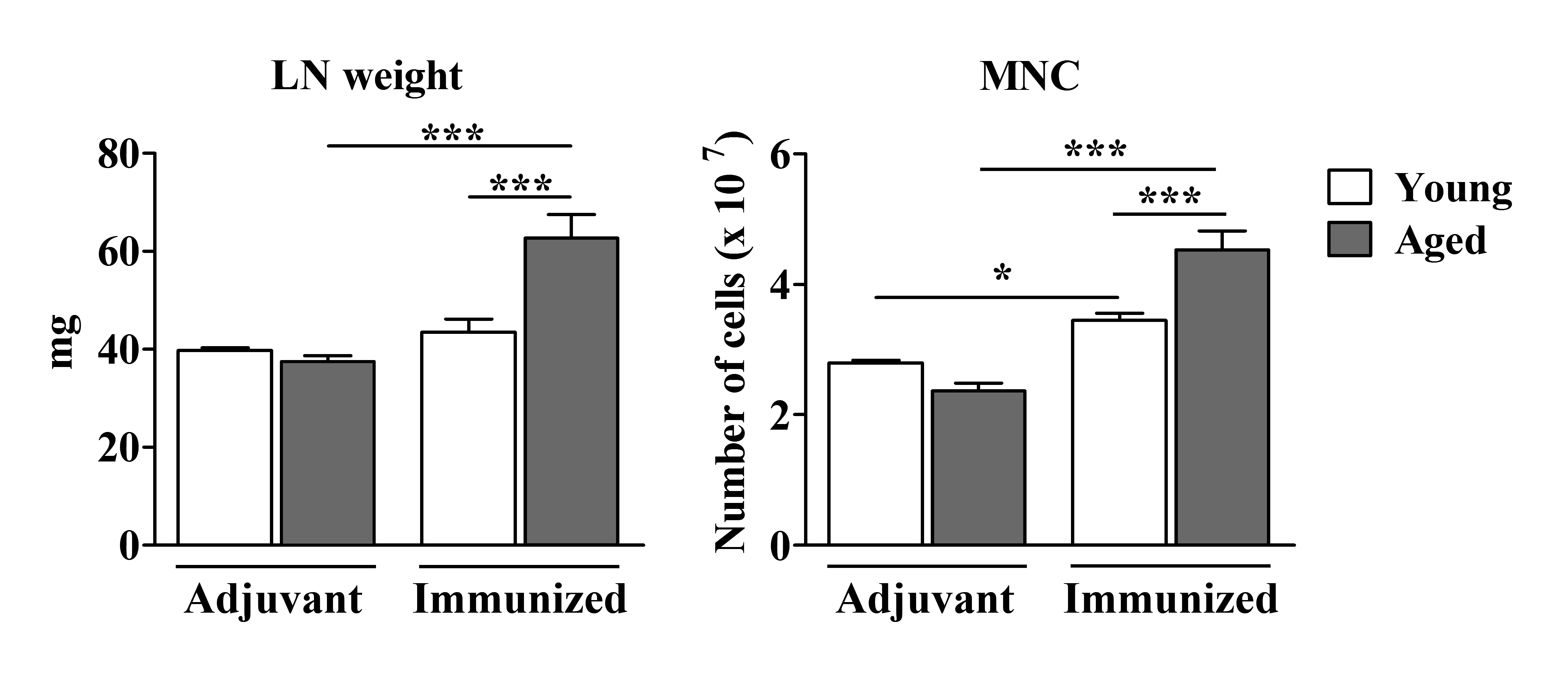

Supplement: Additional file 3: Figure S3. — Aging increases weight and cellularity of draining lymph nodes from AO rats immunized for EAE. Bar graphs indicate the (left) lymph node (LN) weight and (right) number of draining lymph node mononuclear cells (MNC) from young and aged rats injected with CFA and Bordetella pertussis (Adjuvant) or immunized for EAE (Immunized). All results are presented as means ± SEM (n = 9/group). Data are representative of one of two experiments with similar results. *p < 0.05; ***p < 0.001. (TIFF 168 kb) [file 12979_2015_44_MOESM3_ESM.tif]

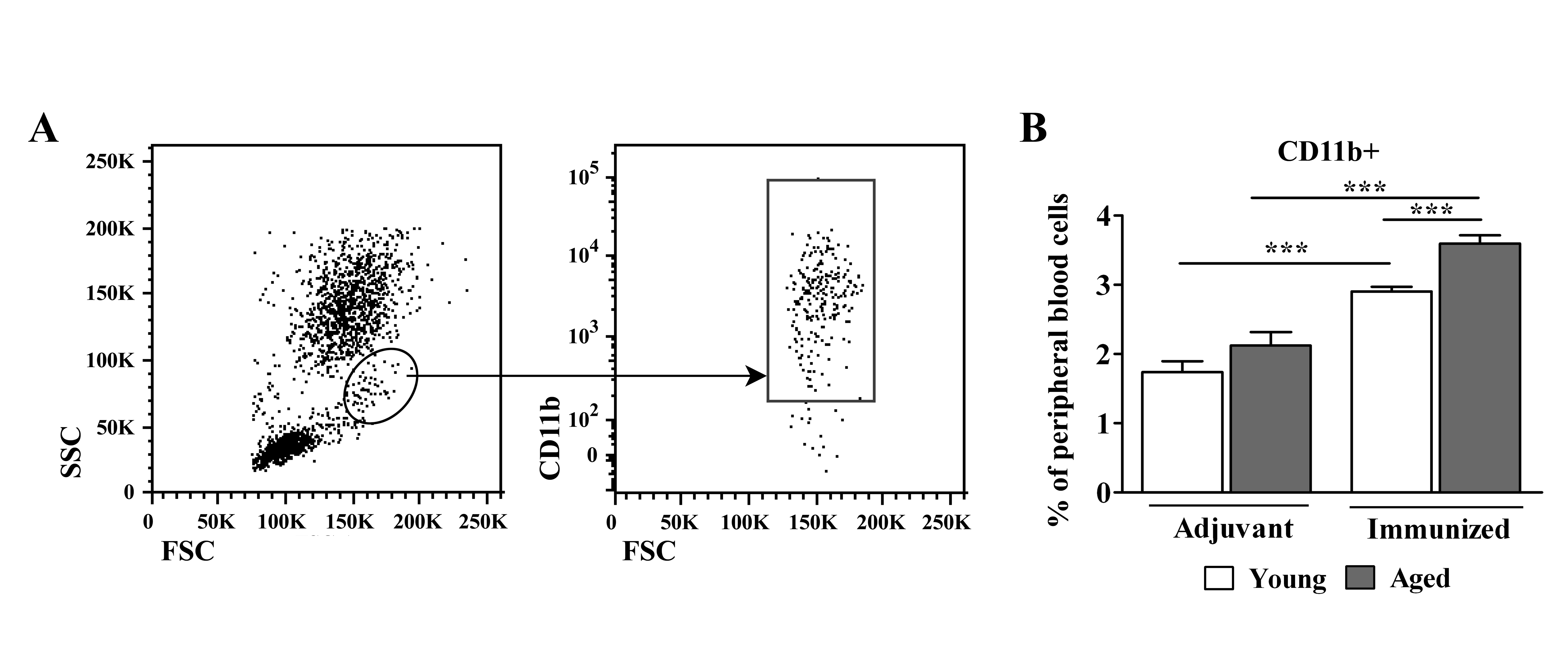

Supplement: Additional file 4: Figure S4. — Aging increases the frequency of CD11b + cells with monocyte/macrophage physical characteristics in peripheral blood from rats immunized for EAE. (Panel A) Flow cytometry dot plots indicate peripheral blood CD11b + monocyte/macrophage gating strategy. Flow cytometry dot plot indicates (right) CD11b expression on monocytes/macrophages gated according to FSC and SSC as shown in (left) flow cytometry dot plot. (B) Bar graph shows percentage of CD11b + monocytes/macrophages within peripheral blood cells from young and aged rats injected with CFA and Bordetella pertussis (Adjuvant) or immunized for EAE (Immunized). All results are presented as means ± SEM (n = 9/group). Data are representative of one of two experiments with similar results. ***p < 0.001. (TIFF 1294 kb) [file 12979_2015_44_MOESM4_ESM.tif]
